# Exploring Herbal Compounds as Targeted Therapies for Breast Cancer: Insights from Network Pharmacology, Molecular Docking, MD Simulation, ADME-Toxicity and DFT Profiles

**DOI:** 10.5812/ijpr-153579

**Published:** 2024-12-25

**Authors:** Haixia Zhang

**Affiliations:** 1 College of Traditional Chinese Medicine, Hebei North University, Zhangjiakou City, 075000, China

**Keywords:** Breast Cancer, Herbal Compounds, Traditional Chinese Medicine, Network Pharmacology, Molecular Docking, MD Simulation

## Abstract

**Background:**

Herbal compounds sourced from various plants are becoming targeted therapies for breast cancer.

**Objectives:**

This study aims to explore the potential of focusing on herbal compounds as targeted therapies for breast cancer using computational techniques.

**Methods:**

A total of 129 herbal compounds linked with breast cancer were identified from the Traditional Chinese Medicine Systems Pharmacology Database and Analysis Platform (TCMSP) database. Molecular docking and MD simulation were carried out against three protein targets linked with breast cancer. Network pharmacology was used to identify the common plant sources for the bioactive compounds, and interaction networks were constructed. The ADME-toxicity profiles and density functional theory (DFT) analysis were calculated for the top docking hits.

**Results:**

Dipiperitylmagnolol and sophoranone were identified as the top docking hits and lead compounds. Network pharmacology analysis revealed *Magnolia species *as the common plant sources having multiple bioactive compounds. MD simulation analysis revealed conformational stability of the top docking hits. The analyses underscore the robust binding potential of dipiperitylmagnolol and its possible therapeutic relevance in targeting breast cancer pathways. ADME-toxicity and DFT analysis provided insights into the pharmacokinetic and electronic behavior of the top docking hit. Combinatorial study of herbal therapies with conventional treatments will increase the therapeutic efficacy for breast cancer treatment.

**Conclusions:**

The study provides insights into the implications of herbal compounds as targeted therapy for breast cancer. Therefore, the study recommends further experimental validation and development of herbal-based compounds for the treatment of breast cancer.

## 1. Background

Breast cancer is a major global health challenge and a leading cause of cancer-related deaths among women. The disease is generally categorized by its heterogeneous hormone receptor-positive and triple-negative subtypes (1). Owing to the heterogeneity of these disease subtypes and the involvement of various signaling pathways, new advanced therapies are necessary. The specific pathway that Traditional Chinese Medicine (TCM) may offer promising compounds in the discovery of more effective agents against breast cancer remains relevant, given its centuries-old foundation as an alternative and holistic tradition of healing (2). Traditional Chinese Medicine has been utilized for thousands of years to treat cancer and other diseases based on the pharmacological properties of herbs, minerals, and animal products. Traditional Chinese Medicine takes a holistic approach and thus treats not only the disease but also focuses on restoring Qi (our body's vital energy), manipulating meridians, and correcting yin-yang imbalances to ensure that natural healing mechanisms are enhanced (3). Therefore, the integration of TCM approaches with the rapid evolution of computational techniques such as computer-aided drug designing (CADD) has a large scope to discover and optimize potent bioactive molecules from traditional Chinese medicine for their therapeutic applications in breast cancer treatment (4). Computer-aided drug designing analysis utilizes compounds and target proteins linked to target diseases for analysis. In the present study, epidermal growth factor receptor (EGFR), Aromatase, and PI3K alpha were chosen as targets as they are key proteins involved in disease progression and metabolic processes in breast cancer. These proteins are common targets in cancer research. Computer-aided drug designing is a rapidly growing discipline that integrates computational chemistry, bioinformatics, and structural biology into the drug discovery process (5). Computer-aided drug designing techniques such as molecular docking, virtual screening, and ADME-toxicity prediction have made it possible to use CADD in predicting the binding affinity of compounds with their protein targets, bioactivity predictions for a given compound, drug candidate selection, and optimization towards pharmacokinetic (ADME) or pharmacodynamic properties (6). The technique is especially useful for the rapid exploration of the extensive chemical space in TCM, as traditional experimental methods can be time-consuming and expensive (7). In this study, we aim to leverage the synergy between TCM and CADD approaches for targeting breast cancer. Using various computational approaches such as molecular docking, molecular dynamics simulations, ADMET, and density functional theory (DFT), we aim to discover and investigate potential leads from TCM sources with prospective anti-breast cancer activities (8). These results, therefore, represent a necessary step in translating centuries-old TCM traditional herbal formulas into new breast cancer precision medicinal candidates and provide valuable CADD-accelerated discovery and development for safe drugs. The present study also aims to explore their molecular mechanisms of action, binding modes, and pharmacokinetic properties to facilitate further clinical drug development (9).

## 2. Methods

### 2.1. Traditional Chinese Medicine Chemical Compounds 

A search was carried out in the Traditional Chinese Medicine Systems Pharmacology Database and Analysis Platform (TCMSP) to retrieve the major chemical compounds associated with breast cancer. The retrieved compounds were meticulously curated to ensure their relevance to breast cancer treatment. The screening was based on the reported pharmacological activities of the compounds, especially their anticancer properties. The analysis identified several compounds with effects on pathways most relevant to human breast cancer, such as cell proliferation, apoptosis, and angiogenesis. The 3D structures of the selected compounds were retrieved from the NCBI PubChem Database, and the details of the compounds are described in Appendix 1 in Supplementary File. These structures were then optimized using standard molecular force fields (MM2) with ChemOffice 2010 (Perkin Elmer, USA).

### 2.2. Target Proteins of Breast Cancer 

Identifying suitable target proteins associated with breast cancer is a crucial step. Target proteins relevant to breast cancer must be chosen based on the availability of structural data and known binding sites. The selection of target proteins with good interaction potential with ligands involved in key breast cancer pathways is essential for effective analysis. Protein targets associated with breast cancer were extracted from the Cancer Drugs Database. Three PDB IDs of breast cancer protein targets were collected from the Protein Data Bank. The proteins are epidermal growth factor receptor kinase (PDB ID: 1XKK), aromatase (PDB ID: 3S7S), and PI3K Alpha (PDB ID: 7PG6). 

Protein targets were selected based on their established roles in breast cancer pathophysiology, druggability, and clinical relevance to current therapeutic strategies. These proteins are targeted with the belief that selective modulation of key regulatory proteins and pathways may lead to the identification of novel bioactive compounds capable of inhibiting breast cancer progression and modulating sensitivity and response phenotypes.

### 2.3. Molecular Docking Study 

After a comprehensive literature review and retrieval of compounds from the database, compounds linked to breast cancer were selected to undergo molecular docking to assess their binding interactions with specific target proteins. This process involves calculating binding scores and analyzing interactions to prioritize candidates with the strongest affinities. 

Molecular docking simulations were carried out against the three protein targets associated with breast cancer, namely epidermal growth factor receptor kinase (PDB ID: 1XKK), aromatase (PDB ID: 3S7S), and PI3K Alpha (PDB ID: 7PG6). MVD 7.0 (Molexus IVS, Denmark) was used for docking, employing a grid-based docking methodology. 

Initially, the binding pockets and active sites of the breast cancer protein targets were identified. Bond flexibility and side chain flexibility were set to standard values (tolerance = 1.0, strength = 0.90). The root mean square deviation (RMSD) threshold was set to 2.00 Å, with the simplex method as the evolution size, and the number of iterations for matching was set to 1,000 iterations. 

Flexible ligands and protein receptors were used, resulting in a realistic simulation using MVD. This approach enabled a more accurate simulation of the binding process, where both the ligand and receptor could undergo conformational changes.

### 2.4. Construction of Interaction Networks 

To elucidate the relationships between bioactive compounds, their plant sources, and the target proteins associated with breast cancer, a network interaction map was constructed. This process involved multiple steps to visualize and analyze the complex interactions within the dataset. 

A network interaction map was created for the top docking hits, and the medicinal plants associated with the target enzymes linked to the breast cancer network were also constructed using Cytoscape version 3.9. The entities in the network were characterized as nodes, while the relationships between them were depicted as edges. The network's structure was analyzed to identify key players and assess biological significance, aiding in uncovering potential therapeutic strategies for breast cancer. 

Each node represented one of three entities: A bioactive compound, a plant source, or a target protein. Edges in the network depicted the relationships between nodes. Three primary types of edges were constructed: Compound-protein edges, plant-compound edges, and compound-compound edges.

### 2.5. MD Simulation 

MD simulation for the protein-ligand binding interactions was conducted using Desmond (Schrödinger Inc). The Protein Preparation Wizard was employed to pre-process the protein-ligand complexes. The System Builder tool was used to prepare the systems with the OPLS_2005 FF, optimized for compatibility with both proteins and protein-ligand complexes. 

A solvent model was used to simulate physiological conditions, employing TIP3P water molecules in an orthorhombic box. The system was neutralized with counter ions and supplemented with 0.15 M NaCl. The orthorhombic box ensures these concentrations are achieved without excessive dilution or molecular crowding. Its adjustable dimensions enable efficient packing of solvent molecules and solutes while optimizing computational resources, striking a balance between accuracy and efficiency. 

The Martyna-Tuckerman-Klein Barostat was utilized to simulate the NPT ensemble at 300 K and 1 atm pressure. MD simulation was run for 100 ns, and the stability of the simulation was evaluated by analyzing protein-ligand contacts and calculating RMSD and root mean square fluctuation (RMSF).

The dynamic cross-correlation between different atoms and residues in the molecular system was analyzed using the dynamic cross-correlation matrix (DCCM). Additionally, the principal components of molecular dynamics simulations were examined using principal component correlation (PCC) to understand the major movements and fluctuations of the molecule.

### 2.6. Absoption, Distribution, Metabolism and Excretion-Toxicity Study 

For the ADME-toxicity study, compounds of interest were selected based on their binding interactions with specific target proteins identified through molecular docking simulations. The top-ranking compounds from the docking results, selected based on binding affinity and interaction profiles with key residues in the protein active site, were subjected to ADME-toxicity analysis to evaluate their pharmacokinetic and safety profiles. 

Absoption, distribution, metabolism and excretion-Toxicity analysis was performed using ADMET-AI, which predicts ADMET properties through a graph neural network architecture called Chemprop-RDKit, and SWISS-ADME. The SWISS-ADME tool was employed to generate the Bioavailability Radar map, which evaluates key parameters, including lipophilicity (LIPO), molecular weight (SIZE), polarity (POLAR), insolubility (INSOLU), insaturation (INSATU), and flexibility (FLEX). 

The biological activities, ADME properties, and toxicity profiles of the top docking hits were comprehensively analyzed. This study utilized a range of computational tools to predict pharmacokinetic and toxicity properties. For each compound in the dataset, the SMILES notation or chemical structure files were used as input. The SMILES notation provides a text-based representation of the chemical structure, enabling accurate computational analysis.

### 2.7. Density Functional Theory Analysis 

Density functional theory calculations were carried out using Gaussian 9.0 (Gaussian, Inc.) for the top docking hits of EGFR (PDB ID: 1XKK), Aromatase (PDB ID: 3S7S), and PI3K Alpha (PDB ID: 7PG6). The calculations were performed at the ground state using the DFT/B3LYP/LanL2DZ level of theory and extended Hückel with mixed HOMO and LUMO orbitals. The band gap energy (ΔE_LUMO-HOMO_) was also determined to understand the electron donor and acceptor properties of the docking hits. This analysis provides insights into the electronic properties and reactivity of the compounds, which are critical for evaluating their potential as therapeutic agents.

## 3. Results

### 3.1. Molecular Docking 

**Figure 1.**
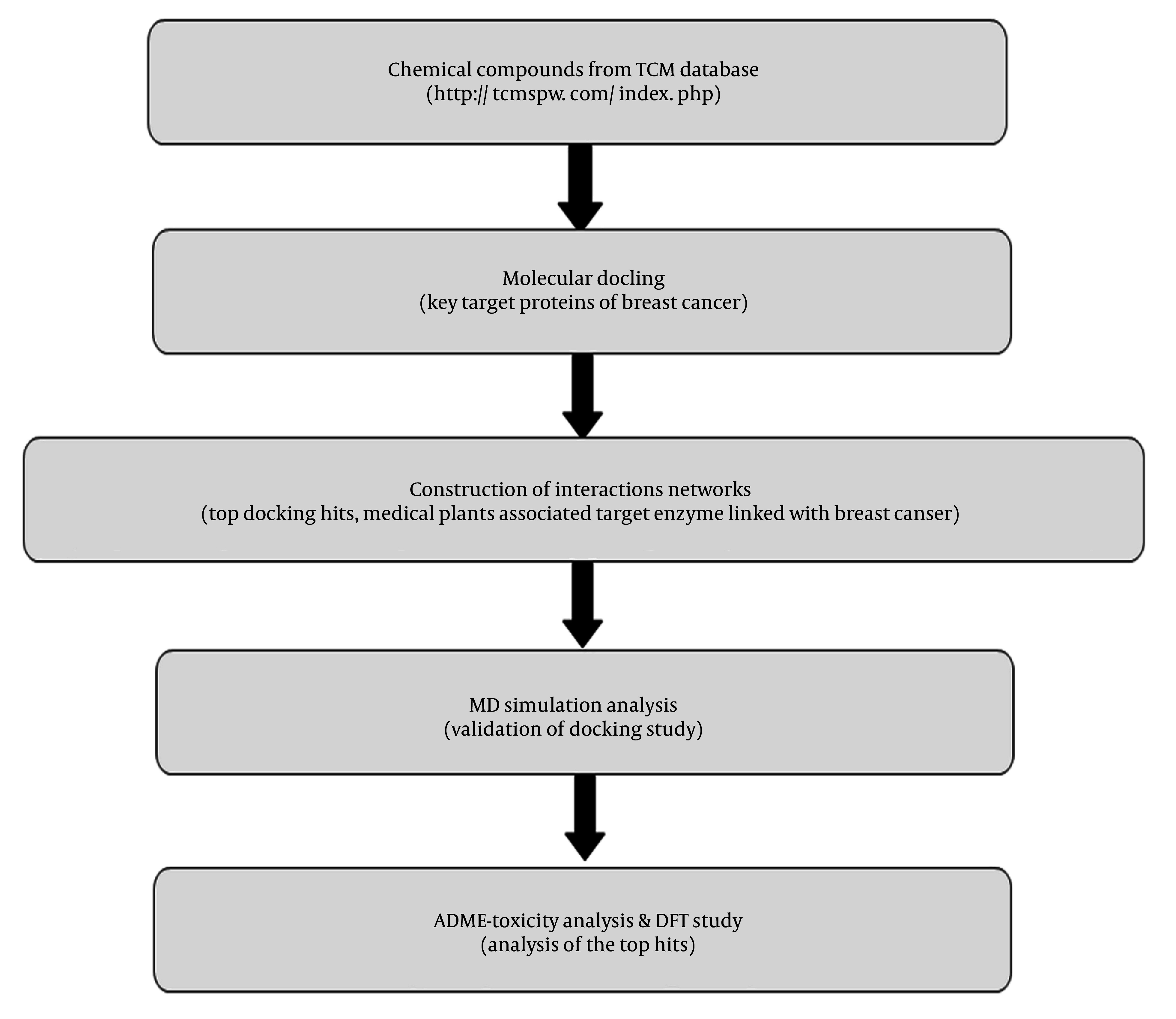
Flow chart showing the workflow of the present study

The workflow detailing the step-by-step description of the present study is presented in Figure 1. The molecular docking simulation results for the target proteins associated with breast cancer are shown in Table 1. For the PDB IDs 1XKK, 3S7S, and 7PG6, dipiperitylmagnolol exhibited the highest MolDock Scores (-134.26, -139.87, and -122.40, respectively) and Rerank scores (-107.12, -92.44, and -97.34, respectively), indicating strong binding affinity at these binding sites (Table 1). 

**Table 1.** Docking Result of 1XKK and 3S7S and 7PG6 (Top 10 Hits and Controls)

Ligand	MolDock Score (kcal/mol)	Rerank Score (kcal/mol)	Total Score (kcal/mol)
**1XKK (EGFR)**			
Dipiperitylmagnolol	-134.3	-107.1	-492.2
Sophoranone	-126.4	-99.4	-465.5
(S)-rosmarinic acid	-118.7	-110.2	-457.0
Sophoradochromene	-116.4	-97.3	-451.6
Gamma-tocotrienol	-111.9	-96.9	-440.1
Alnustone	-108.9	-96.8	-436.0
Mulberrofuran A	-115.5	-96.6	-434.7
Piperitylhonokiol	-116.4	-96.4	-434.2
Eurycarpin A	-109.0	-100.7	-432.1
Moracin D	-114.8	-95.4	-423.6
Lapatinib (control)	-96.9	-90.3	-403.3
**3S7S (aromatase)**			
Dipiperitylmagnolol	-139.9	-92.4	-495.3
Sophoranone	-141.6	-101.1	-493.9
Sophoradochromene	-125.3	-105.9	-473.7
Licoagroisoflavone	-117.3	-105.3	-455.0
Piperitylhonokiol	-117.1	-98.5	-450.3
Gamma-tocotrienol	-116.2	-94.5	-440.3
Moracin H	-115.5	-99.9	-435.9
Phaseol	-117.1	-95.6	-424.8
Dihydronitidine	-110.8	-93.1	-421.4
Phaseollinisoflavan	-111.2	-91.1	-419.5
Exemestane (control)	-109.1	-91.9	-399.9
**7PG6 (PI3K alpha)**			
Dipiperitylmagnolol	-122.4	-97.3	-453.8
Arctigenin	-118.3	-96.0	-427.2
Gingerenone A	-106.2	-94.3	-419.6
Phaseol	-109.7	-92.5	-416.1
Gingerenone B	-100.3	-85.1	-413.4
Gingerenone C	-101.9	-84.2	-402.8
Lupiwighteone	-94.3	-88.6	-399.2
Isolicoflavonol	-101.5	-90.8	-398.9
Wedelolactone	-102.6	-88.5	-396.6
Glycyrol	-106.5	-90.9	-396.6
Alpelisib	-98.8	-80.9	-380.4

Abbreviation: EGFR, epidermal growth factor receptor.

Dipiperitylmagnolol demonstrated superior binding to the 1XKK protein compared to lapatinib, with significantly higher MolDock, interaction, and total scores (-492.17 vs. -403.29 kcal/mol; Appendix 2 in Supplementary File). Similarly, it exhibited higher binding affinity to the 3S7S protein than exemestane, as reflected in its more favorable MolDock, interaction, docking, and total scores (-495.26 vs. -399.89 kcal/mol; Appendix 3 in Supplementary File). For the 7PG6 protein, dipiperitylmagnolol also showed stronger binding than alpelisib, based on MolDock, rerank, interaction, and total scores (-453.84 vs. -380.43 kcal/mol; Appendix 4 in Supplementary File). These findings highlight dipiperitylmagnolol as a promising candidate for further therapeutic research, showing the highest binding affinity with aromatase (3S7S), EGFR (1XKK), and PI3K alpha (7PG6) with the lowest Total Scores of -495.26, -492.17, and -453.84, respectively. This confirms its robust binding potential at these sites, as summarized in Table 1. 

Sophoranone also displayed favorable binding affinity with aromatase (3S7S) and EGFR (1XKK), with Total Scores of -493.86 and -465.46, respectively (Table 1). 

This analysis explores the binding affinities of these ligands against key proteins involved in cancer pathways: Epidermal growth factor receptor (EGFR, PDB ID: 1XKK), aromatase (PDB ID: 3S7S), and Phosphoinositide 3-kinase alpha (PI3K alpha, PDB ID: 7PG6). These ligands fall under pharmacological groups such as coumarins, phenols, and flavonoids, which impart significant anti-inflammatory, antioxidant, and anticancer activities. 

The structural features of these pharmacological groups are pivotal for the biological activities of the ligands, influencing their interactions with various biological targets, including enzymes. Since these proteins play crucial roles in cancer development and progression, they are significant targets for drug discovery. Detailed energy scores and docking scores for 1XKK, 3S7S, and 7PG6 are provided in Appendices 2 - 4 in Supplementary File, respectively. 

### 3.2. Molecular Interaction Analysis 

**Figure 2.**
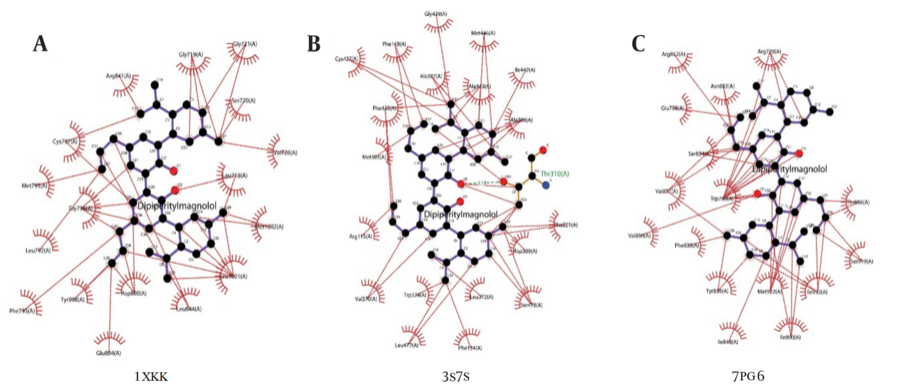
Molecular interaction map of the top docking hit (dipiperitylmagnolol) at the active site of 1XKK, 3S7S and 7PG6. The docking results showed the binding affinities between dipiperitylmagnolol and the protein active sites (1XKK, 3S7S and 7PG6), highlighting key interactions such as hydrogen bonding, hydrophobic contacts, and van der Waals interactions.

The molecular interaction analysis of the top hits, which elucidates the specific interactions between ligands and target molecules, is presented in Figure 2. The interaction analysis highlights that dipiperitylmagnolol and sophoranone demonstrate significant potency across multiple targets. The molecular interaction maps of dipiperitylmagnolol at the active sites of 1XKK (EGFR Kinase), 3S7S (Aromatase), and 7PG6 (PI3K alpha) are shown in Figure 2A - C, respectively. 

The energy map analysis, depicting the hydrogen bond acceptor (HBA)/hydrogen bond donor (HBD) regions and electropositive/negative regions of dipiperitylmagnolol at the active sites of 1XKK, 3S7S, and 7PG6, is presented in Appendix 8A, B, and C in Supplementary File. Appendix 1 in Supplementary File further provides a detailed energy map interaction analysis, where HBA and HBD regions are illustrated in yellow and turquoise colors, respectively. 

The energy map analysis reveals important ligand-protein interaction regions and highlights areas with favorable electrostatic complementarity. Hydrogen bond acceptor regions (yellow) correspond to protein atoms or functional groups that accept hydrogen bonds from donor atoms on the ligand, while HBD regions (turquoise) correspond to atoms in the protein capable of donating hydrogen bonds to ligand acceptors. Adjusting HBD and HBA properties can improve the stability, selectivity, and bioavailability of a drug by enhancing its binding affinity and specificity. 

Appendix 9 in Supplementary File presents the electrostatic interaction map of the top docking hit, dipiperitylmagnolol, at the active sites of 1XKK (Appendix 9A in Supplementary File), 3S7S (Appendix 9B in Supplementary File), and 7PG6 (Appendix 9C in Supplementary File). The electrostatic analysis identifies regions of charged atoms or groups (positive or negative) on both the drug and protein targets. These charged regions are critical for forming electrostatic interactions, which significantly strengthen the binding between the drug and the target protein. 

This comprehensive analysis provides detailed insights into ligand binding affinities and protein-ligand interactions. It identifies dipiperitylmagnolol as a lead compound with robust binding potential to 1XKK, 3S7S, and 7PG6, suggesting its promising therapeutic potential as a EGFR, aromatase, and PI3K alpha inhibitor.

### 3.3. Construction of Interaction Networks 

**Figure 3.**
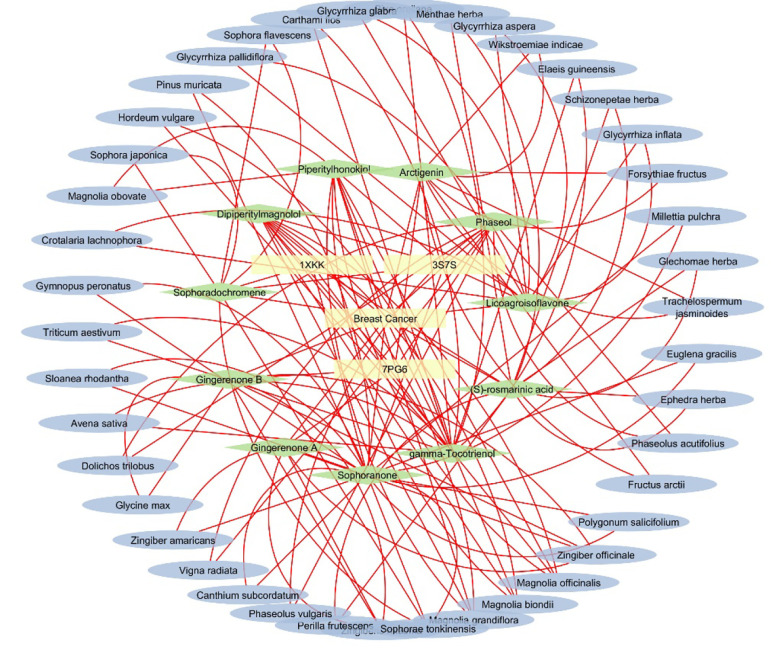
Network pharmacology analysis of the top 3 hits herbal compounds along with their respective source plant source. The map provides a holistic herbal compounds interactions identifying key targets, pathways, and biological processes involved in breast cancer, offering insights into the mechanism of action for potential therapies.

The details of the herbal compounds associated with breast cancer are presented in Appendix 1 in Supplementary File. These compounds represent a rich array of bioactive molecules with potential therapeutic relevance to breast cancer. The network pharmacology map of the investigated compounds, their target molecules, and pathways, illustrating the relationships between various elements linked to breast cancer, is provided in Appendix 10 in Supplementary File. The plant sources of the top five docking hits for 1XKK, 3S7S, and 7PG6 are detailed in Appendix 5 in Supplementary File. The interaction network of the top three hit compounds, their plant sources, and their target proteins, generated using Cytoscape, is presented in Figure 3. The results revealed that the network map uncovered key protein targets and their associated herbal compound plant sources. Nodes with a high degree of connectivity, known as hubs, were identified, including pivotal proteins such as 1XKK (EGFR Kinase), 3S7S (aromatase), and 7PG6 (PI3K alpha), which interact with multiple bioactive compounds, indicating their central roles in breast cancer pathways. 

Figure 3 represents the network interaction analysis of the top three docking hits (herbal compounds) for 1XKK, 3S7S, and 7PG6, along with their respective plant sources. Additionally, Appendix 11 in Supplementary File provides the network interaction map of the top three docking hits for 1XKK, 3S7S, and 7PG6, respectively. 

Notably, species from the *Magnolia* genus were highlighted for their ability to produce multiple bioactive compounds with significant therapeutic potential (Appendix 11 in Supplementary File). Among these, sophoranone and gamma-tocotrienol were the most prominent herbal compounds, being associated with nine other bioactive compounds. 

The present investigation identified the top docking hits from *Magnolia biondii*, *Magnolia grandiflora*, and *Magnolia officinalis*. These compounds include dipiperitylmagnolol, a bisphenolic derivative of magnolol containing two piperityl groups attached to the magnolol backbone. This prevalence underscores the importance of these plants in the context of breast cancer therapeutics and highlights their potential for further research and development of novel therapeutic agents. 

The findings emphasize the significance of the *Magnolia* genus in traditional Chinese medicine as a valuable source for bioactive compounds with anti-cancer properties.

### 3.4. MD Simulation Analysis 

**Figure 4.**
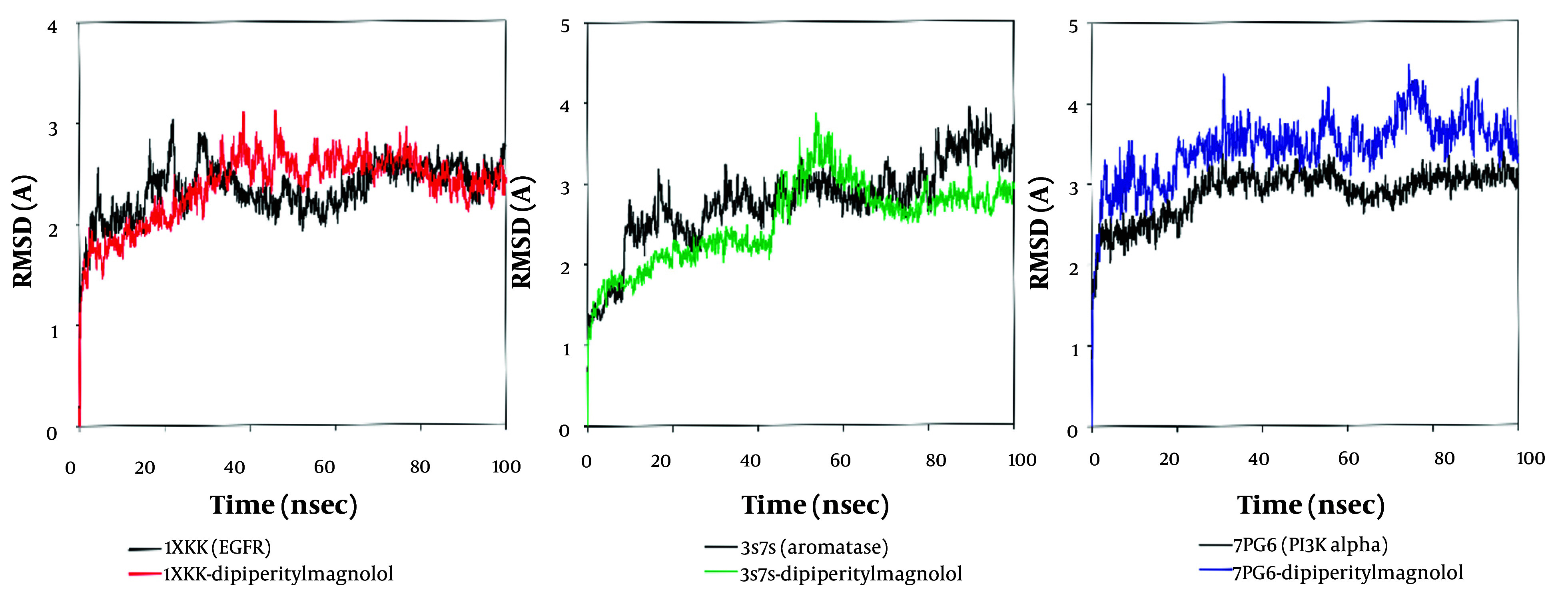
A, root mean square deviation (RMSD) analysis of 1XKK-dipiperitylmagnolol; B, 3S7S-dipiperitylmagnolol; and C, 7PG6-dipiperitylmagnolol during the 100 ns MD production. The graph shows a stable RMSD which indicates that dipiperitylmagnolol is well-bound to the active site of the proteins (1XKK, 3S7S and 7PG6) = maintaining its conformation throughout the simulation, implying a potential stable inhibitor.

Figure 4 presents the RMSD analysis of 1XKK-dipiperitylmagnolol (Appendix 12A in Supplementary File), 3S7S-dipiperitylmagnolol (Appendix 12B in Supplementary File), and 7PG6-dipiperitylmagnolol (Appendix 12C in Supplementary File) during the 100 ns MD production. The RMSD gives an estimate of the change in the displacement of the atoms during the 100 ns time frame, providing insight into its structural conformation. The graph provides insights into the structural stability and binding of the ligand to the target protein from 0 ns to 100 ns. The RMSD values from the graph indicate that the corresponding ligand binding mode with the respective protein conformation is more stable. 

Appendix 12 in Supplementary File presents the amino acid-ligand interaction and its timeline representation monitored during the 100 ns MD production for 1XKK-Dipiperitylmagnolol (Appendix 12A), 3S7S-dipiperitylmagnolol (Appendix 12B in Supplementary File), and 7PG6-Dipiperitylmagnolol (Appendix 12C in Supplementary File). The figure provides the H-Bonds, Hydrophobic, Ionic, and Water Bridges contacts the protein makes with the ligand during the course of the trajectory. 

The RMSF, which characterizes the shifts and variations of the protein and the protein-ligand complex during the 100 ns MD simulation, is presented in Appendices 13 - 15 in Supplementary File for 1XKK, 3S7S, and 7PG6, respectively. 

The protein secondary structure elements (SSE) of 1XKK and 1XKK-dipiperitylmagnolol complex; 3S7S and 3S7S-dipiperitylmagnolol complex; and 7PG6 and 7PG6-dipiperitylmagnolol complex are presented in Appendices 16 - 18, respectively, in Supplementary File. These plots provide an estimate of the SSE composition for each trajectory frame during the 100 ns simulation. 

**Figure 5.**
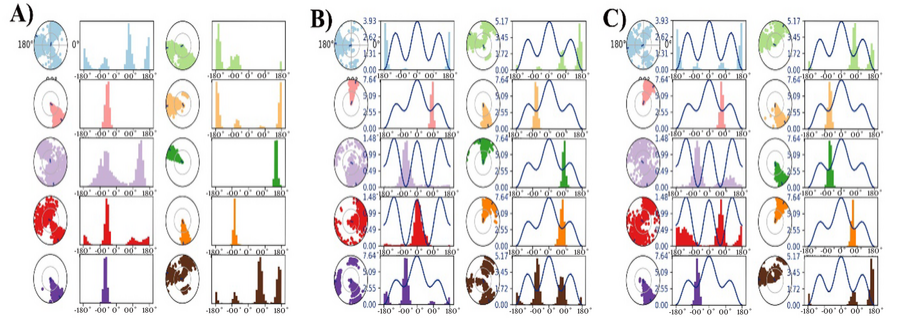
A, torsion profile of the plot of 1XKK-dipiperitylmagnolol; B, 3S7S-dipiperitylmagnolol; and C, 7PG6-dipiperitylmagnolol. The plot summarizes the conformational evolution of every rotatable bond (RB) in the ligand throughout the simulation trajectory from 0 ns to 100 ns.

Figure 5 presents the ligand torsions plot of 1XKK-dipiperitylmagnolol (Figure 5A), 3S7S-dipiperitylmagnolol (Figure 5B), and 7PG6-Dipiperitylmagnolol (Figure 5C), summarizing the conformational evolution of every rotatable bond (RB) in the ligand during the MD production.

**Figure 6.**
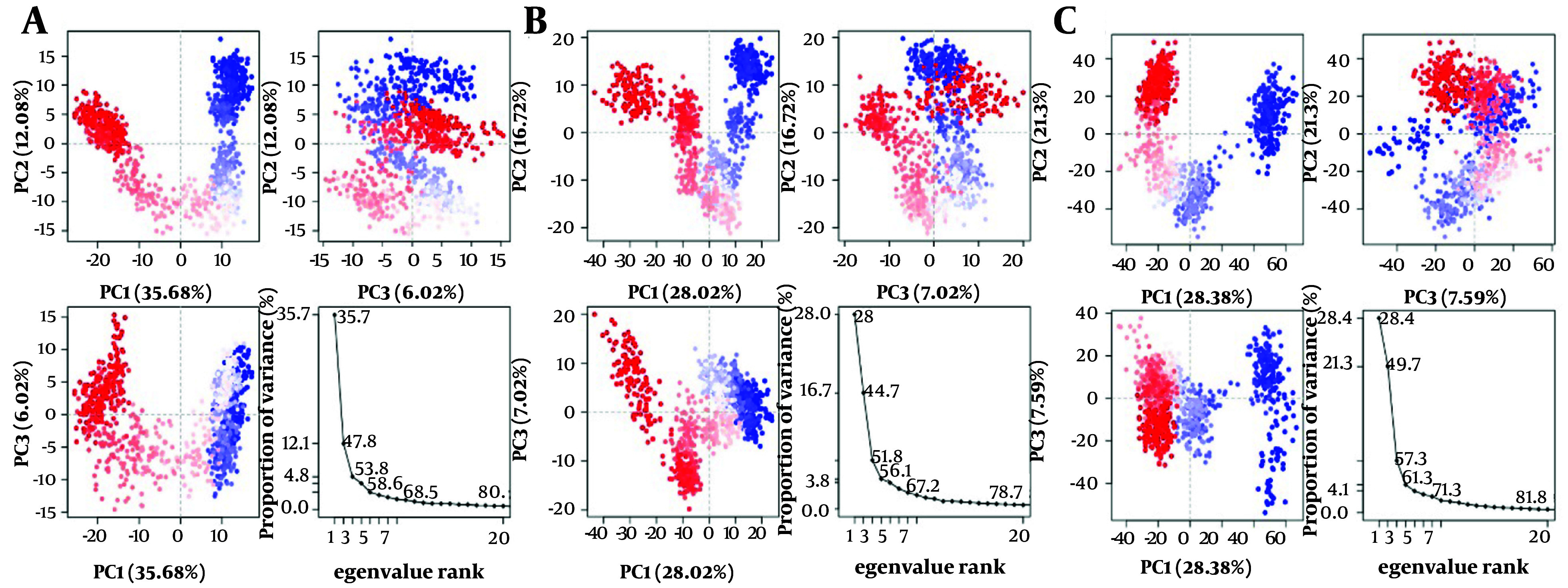
A, principal component correlation (PCC) plot of 1XKK-dipiperitylmagnolol; B, 3S7S-dipiperitylmagnolol; and C, 7PG6-dipiperitylmagnolol during the 100 ns MD production. The plot provides insights into the correlation between protein-ligand interaction components and its binding free energy.

Furthermore, the ligand atom interaction profiles of the 1XKK-Dipiperitylmagnolol complex, 3S7S-Dipiperitylmagnolol complex, and 7PG6-Dipiperitylmagnolol complex at the active site of 1XKK, 3S7S, and 7PG6 are presented in Appendices 19 - 21 in Supplementary File respectively. These analyses provide insights into the dynamic nature of binding interactions of dipiperitylmagnolol at the active site of these proteins. Appendix 22 in Supplementary File presents the DCCM plot analysis of 1XKK-Dipiperitylmagnolol (Appendix 22A in Supplementary File), 3S7S-dipiperitylmagnolol (Appendix 22B in Supplementary File), and 7PG6-dipiperitylmagnolol (Appendix 22C in Supplementary File) during the 100 ns MD production. The plot revealed the dynamic cross-correlation between different atoms and residues of the docked molecular system. It also gives an estimate into the correlated movements of atoms and residues during MD simulations, which is crucial for studying the conformational changes, stability, and interactions. Figure 6 presents the PCC Plot of 1XKK-Dipiperitylmagnolol (Figure 6A), 3S7S-Dipiperitylmagnolol (Figure 6B), and 7PG6-Dipiperitylmagnolol (Figure 6C) during the 100 ns MD production. The PCC plot provides the overall dynamics of the protein-ligand complex and helps in identifying key conformational changes, structural changes, and complex molecular behaviors. It also helps in understanding the major modes of motion in a system and how these modes correlate with each other.

### 3.5. Absoption, Distribution, Metabolism and Excretion-Toxicity Analysis 

**Figure 7.**
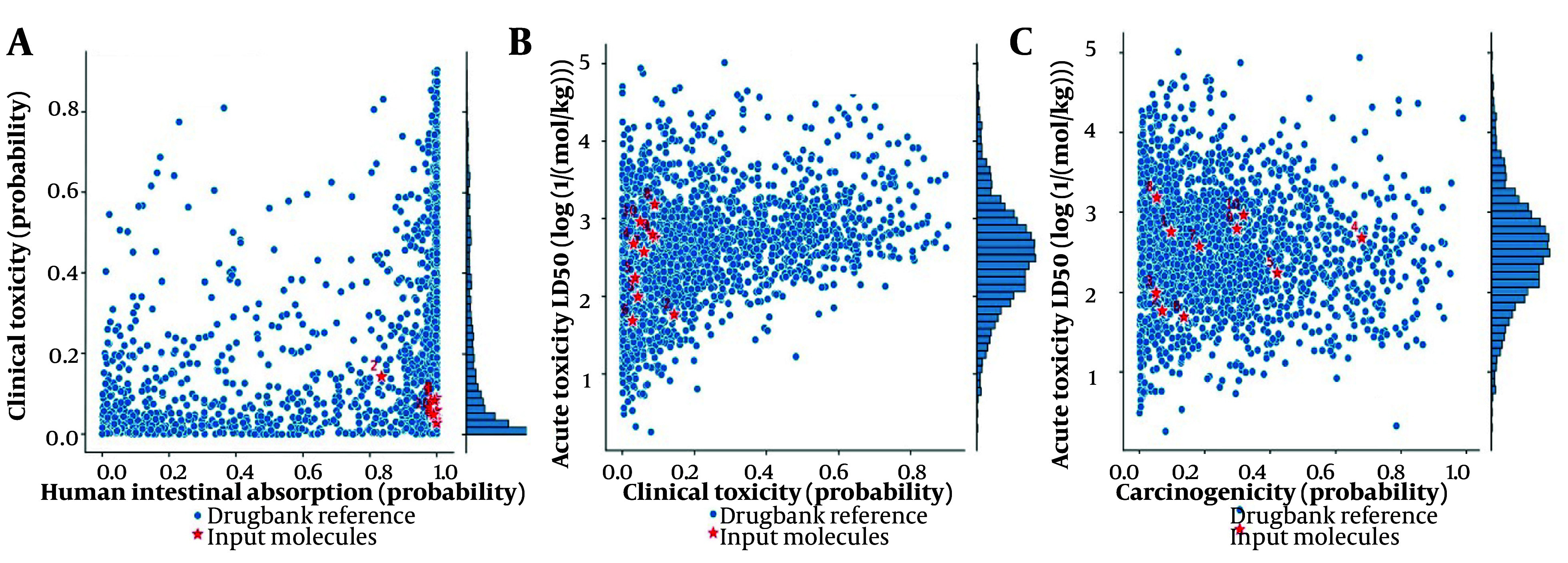
A, ADME-toxicity analysis based on human intestinal absorption; vs B, clinical toxicity clinical toxicity; vs C, acute toxicity lethal dose (LD50) carcinogenicity vs acute toxicity LD50 of the top 5 docking hits of each of the target proteins. (Dipiperitylmagnolol, sophoranone, arctigenin, -(S)-rosmarinic acid, sophoradochromene, gingerenone A, gamma-tocotrienol, piperitylhonokiol, Licoagroisoflavone, phaseol and gingerenone B)

The ADME-Toxicity analysis based on human intestinal absorption vs clinical toxicity (Figure 7A), clinical toxicity vs acute toxicity lethal dose (LD50) (Figure 7B), and carcinogenicity vs acute toxicity LD50 (Figure 7C) suggests that the top docking hits of each of the 3 Proteins lie within the permissible range of all approved FDA drugs. The pharmacokinetic properties from Figure 6 provide an in-depth assessment of the suitability of the investigated compounds for oral administration, their bioavailability, and their potential for metabolism and excretion. The ADME bioavailability radar is also presented in Appendix 23 in Supplementary File.

The knowledge of the ADME properties, as well as toxicological risks, are an essential part of criteria assessment for molecules to be successful in further development toward potential drug candidates. Appendix 6 in Supplementary File reveals the varying levels of drug-likeness, bioavailability, and safety profiles of the top hits. Dipiperitylmagnolol had a drug-likeness (QED) of 0.312 (below 0.5, moderate), bioavailability of 0.65 (above 0.55, good absorption), and an AMES value of 0.18 (below 0.5, non-mutagenic). It also has CYP2C9 inhibition of 0.46 (moderate, potential drug interaction). Overall, dipiperitylmagnolol showed good bioavailability and low clinical toxicity, with moderate enzyme inhibition (Appendix 6 in Supplementary File). 

Appendix 7 in Supplementary File evaluates the toxicity parameters of the top 5 docking hits based on several key indicators such as skin sensitization reaction (SR)-HSE, hERG inhibition (cardiotoxicity), caco-2 permeability, LD50, etc. Dipiperitylmagnolol showed good caco-2 permeability (-5.41) and excellent hydration properties (-3.48), suggesting good intestinal absorption and solubility (least negative values, indicating better hydration and solubility properties). 

### 3.6. Density Functional Theory Analysis 

Appendix 24 in Supplementary File depicts the HOMO (S17A) and LUMO (S17B) energies of the top docking hit dipiperitylmagnolol. The HOMO regions indicate the parts of the molecule where the HOMO has significant electron density, HOMO = -0.03384 eV (Appendix 24A in Supplementary File). Whereas the LUMO is the orbital that will accept electrons if the molecule is excited, LUMO = -0.22121 eV (Appendix 24B in Supplementary File). The band gap energy (Δ_ELUMO-HOMO_) analysis, which involves studying the energy difference between the HOMO and LUMO, is -0.18737 eV. This energy gap is crucial for understanding the electronic properties of materials, as it determines their electrical conductivity and optical behavior. 

## 4. Discussion

The present study combines the rich knowledge of TCM with modern computational methods to identify potential drug candidates and develop novel therapeutic agents for breast cancer treatment (10). Many TCM formulations and herbs have been used for centuries to treat various ailments, including cancer. Today, with the advent of modern computational techniques, researchers are analyzing compounds from these plants and understanding their probable mechanisms for treatment in breast cancer (11). Computer-aided drug designing studies generally use virtual screening and molecular docking techniques to predict active molecules from the vast Chinese medicine database that can bind with selected targets of breast cancer. Simulating the binding of TCM compounds to target proteins allows researchers to identify lead-like natural pseudo-ligands. Typically, Traditional Chinese Medicine treats health and disease in terms of whole system affinities rather than a single-target approach (12). However, molecular docking and virtual screening have their limitations. Relying completely on static protein structures is one of the setbacks of molecular docking, as it may not accurately represent biological conditions. Solvation effects and protein flexibility are often neglected, and scoring functions can be inaccurate. Virtual screening methods may also fail to fully interpret the complex interactions occurring in a cellular environment. Moreover, molecular docking cannot assure bioactivity without subsequent experimental validation. 

Computer-aided drug designing studies are conducted to identify potential TCM compounds and formulate them for targeting multiple breast cancer targets, which may lead to superior therapeutic efficacy (13). Computer-aided drug designing has significant potential to discover and optimize potent bioactive molecules from Traditional Chinese Medicine for their therapeutic applications in breast cancer treatment (4). The results of this study and the analysis highlight the binding affinities of these ligands against key proteins involved in cancer pathways: Epidermal growth factor receptor (EGFR, PDB ID: 1XKK), aromatase (PDB ID: 3S7S), and phosphoinositide 3-kinase alpha (PI3K alpha, PDB ID: 7PG6). In the present study, the docking results for dipiperitylmagnolol across three different proteins (1XKK, 3S7S, and 7PG6) consistently show stronger binding affinity compared to control ligands (lapatinib, exemestane, and alpelisib). The stronger MolDock scores, interaction energies, and total scores for dipiperitylmagnolol indicate that it forms more stable and favorable interactions with these proteins. In contrast, while control ligands demonstrate good binding, they are generally outperformed in terms of overall binding strength.

Dipiperitylmagnolol's higher binding affinity may offer potential therapeutic advantages, as stronger overall interactions can contribute to more effective inhibition of the target proteins. Similarly, Dzobo employed several computational approaches, including molecular docking, molecular dynamics simulations, ADMET, and DFT, to identify and examine potential leads from TCM sources with prospective anti-breast cancer activities (8). Shimu also applied various computational methods, such as molecular docking, molecular dynamics simulations, and ADMET analysis, to evaluate TCM-derived compounds with potential anti-breast cancer activity. Their study focused on identifying lead compounds from TCM sources through virtual screening and molecular docking, similar to the approach used in this study (14). 

Moreover, the potential of plant-derived compounds in cancer treatment has been widely recognized. This is due to the ability of these natural compounds to interfere with cancer progression through various mechanisms, including apoptosis, angiogenesis, and the inhibition of cancer-related enzymes such as aromatase and PI3K (15). Dipiperitylmagnolol's higher binding affinity may offer potential therapeutic advantages, as stronger overall interactions can contribute to more effective inhibition of the target proteins. Similarly, Dzobo employed several computational approaches, including molecular docking, molecular dynamics simulations, ADMET, and DFT, to identify and examine potential leads from TCM sources with prospective anti-breast cancer activities (8). Shimu also applied various computational methods, such as molecular docking, molecular dynamics simulations, and ADMET analysis, to evaluate TCM-derived compounds with potential anti-breast cancer activity. Their study focused on identifying lead compounds from TCM sources through virtual screening and molecular docking, similar to the approach used in this study (14). Moreover, the potential of plant-derived compounds in cancer treatment has been widely recognized. This is due to the ability of these natural compounds to interfere with cancer progression through various mechanisms, including apoptosis, angiogenesis, and the inhibition of cancer-related enzymes such as aromatase and PI3K (15). Plants have long been recognized as a rich source of bioactive components responsible for various medicinal properties (16). In the present study, we examined numerous plant-derived compounds and their anti-aging benefits. Given that thousands of bioactive compounds are present in different plants, the potential for developing new pharmaceuticals and natural remedies is immense (17). These findings pave the way for future research exploring the therapeutic potential of these compounds in pharmaceuticals and healthcare (18). The present study highlights the importance of plant-derived bioactive compounds and their medicinal uses, which can further drive additional research in the fields of natural medicine and drug discovery. Out of the 129 herbal compounds analyzed, dipiperitylmagnolol demonstrated superior performance at multiple binding sites of three different protein targets of breast cancer. As a result, dipiperitylmagnolol was identified as the most promising candidate for multiple protein binding sites (1XKK, 3S7S, and 7PG6), making it a strong drug candidate for further investigation in breast cancer therapy (19). Additionally, sophoradochromene and sophoranone exhibited robust binding affinities at specific protein binding sites. The study also identified a range of compounds with potential therapeutic applications for breast cancer, including sophoradochromene, sophoranone, and arctigenin, which consistently showed favorable docking scores across multiple sites. This information can guide researchers in selecting ligands for specific binding sites to facilitate structure-based design of targeted drugs or treatments. Notably, the study revealed that *Magnolia species,* including *M. officinalis*, are significant sources of bioactive compounds with high affinities toward breast cancer proteins, underscoring their therapeutic potential (20). Thought to exert both anti-inflammatory and antiviral activity, *Magnolia species* have been widely used in TCM. Indeed, it is a folk remedy for many conditions, including cancer, which presumably reflects its anticancer potential. Compounds derived from *Magnolia species *have been demonstrated to modulate many of the same signaling pathways involved in cancer progression, making them compelling leads for further investigation (21). 

*Magnolia species *have been identified as the most common plant sources for many compounds in our dataset, likely indicating that these plants are a rich source of bioactive molecules with potential therapeutic implications for breast cancer. Furthermore, it is worth emphasizing the importance of screening different plants to discover new therapeutic agents, as other species belonging to the *Magnolia* genus may yield specific bioactive compounds. 

The provided data could facilitate the selective isolation and production of bioactive compounds from these plant resources, potentially contributing to the development of new breast cancer treatments. Another important aspect of this research is the construction of interaction networks. These networks illustrate the connectivity between bioactive compounds, their plant sources, and target proteins, providing a comprehensive view of the TCM pharmacopoeia (22). The construction of these interaction networks highlights the links among top-hit compounds, plant sources, and target proteins. Understanding these intricate networks offers a structural basis for the action of compounds on proteins, identifying essential proteins and their associated plant sources, and presenting a complete dataset perspective. 

These networks can be used by researchers to explore potential synergistic effects or combinatorial treatments between different compounds and plant sources. Additionally, the data from the ADME-Toxicity study were crucial for understanding the pharmacokinetic properties and potential safety hazards of these compounds. This information provided a foundation for prioritization in preclinical rodent toxicity-testing programs and informed subsequent clinical development strategies for selected breast cancer therapeutics (23). 

The ADME-Toxicity analysis in this study demonstrated that the top docking hits for the three target proteins fall within the acceptable limits for FDA-approved drugs, underscoring their potential as promising drug candidates. Dipiperitylmagnolol, in particular, exhibits good bioavailability and a low toxicity profile. It has a QED of 0.312, indicating moderate drug-likeness. Although this value is below the optimal threshold of 0.5, its bioavailability of 0.65 exceeds the 0.55 threshold, suggesting adequate absorption. This bioavailability, combined with its low clinical toxicity (0.03), positions dipiperitylmagnolol as a highly interesting candidate for further investigation. The toxicity analysis of dipiperitylmagnolol reveals that it has a unique safety and pharmacokinetic profile, enhancing its potential as a drug candidate. Dipiperitylmagnolol demonstrates a longer half-life and exhibits good caco-2 permeability (-5.41) along with excellent hydration properties (-3.48), indicating favorable intestinal absorption and solubility. Understanding these ADME properties and associated toxicological risks is crucial for advancing these molecules in drug discovery. 

A systematic application of this methodology provides a comprehensive assessment of compound effectiveness and toxicity, which is critical for the bench-to-bedside translation of potential drugs. While CADD studies show great promise for the application of TCM in breast cancer therapy, several challenges need to be addressed before these methods can be fully embraced. These include rigorous experimental validation and standardization of the formulations used (24). In addition, these results underscore the relatively limited understanding of the bioavailability and pharmacokinetic nature of TCM compounds, which must be further explored to facilitate their clinical translation. The DFT analysis highlights the molecular regions where the HOMO and LUMO exhibit significant electron density, offering insights into their electronic properties. However, experimental validation remains a key area requiring greater focus, beyond merely validating control statistics and establishing quality standards, which are essential steps for making TCM-based drugs credible and reliable. While CADD studies effectively identify candidate lead molecules, their translation to clinical use necessitates thorough pharmacokinetics and pharmacodynamics analyses. The ultimate steps in validating TCM-based breast cancer therapies involve comprehensive safety and efficacy assessments through clinical trials. Such a holistic approach will support broader efforts to identify new treatments for breast cancer, reinforcing the potential of plant-derived compounds and paving the way for further investigations. The combinatorial use of herbal therapies with conventional treatments holds promise for enhancing therapeutic efficacy in breast cancer treatment. These findings provide a foundation for developing innovative treatments that could offer hope to patients and their families affected by breast cancer. However, certain limitations of this study must be acknowledged. The reliance on network pharmacology may oversimplify the complex biological interactions involved in breast cancer. Additionally, molecular docking results, while insightful, may not fully capture in vivo conditions or provide an accurate reflection of physiological responses. Furthermore, the lack of experimental studies to confirm the efficacy of the herbal compounds presents a significant gap, highlighting the need for more robust experimental validation to bridge the gap between computational predictions and clinical applicability.

### 4.1. Conclusions

This study concludes that dipiperitylmagnolol is a potent anti-cancer candidate for breast cancer therapy. The docking studies demonstrated the most favorable docking scores and binding affinities across three targeted breast cancer proteins. *Magnolia species *were identified as significant sources of bioactive compounds with substantial therapeutic potential. 

A combinatorial approach that integrates herbal therapies with conventional treatments could enhance therapeutic efficacy by leveraging unique mechanisms of action to improve patient outcomes. This synergy may reduce side effects, enhance drug absorption, and address the challenge of disease resistance, offering a novel strategy for breast cancer management. 

Therefore, the study strongly recommends the exploration and use of herbal compounds for targeted breast cancer treatment.

ijpr-23-1-153579-s001.pdf

## Data Availability

The data presented in this study have been uploaded as a supplementary file during submission and are openly available to readers upon request.
