# Supplementary material for: Exploring Herbal Compounds as Targeted Therapies for Breast Cancer: Insights from Network Pharmacology, Molecular Docking, MD Simulation, ADME-Toxicity and DFT Profiles
Source: Iran J Pharm Res. 2024 Dec 25;23(1):e153579. doi: 10.5812/ijpr-153579 (PMC11892757; doi:10.5812/ijpr-153579)
Supplement: ijpr-23-1-153579-s001.pdf [file ijpr-23-1-153579-s001.pdf]

Exploring Herbal Compounds as Targeted Therapies for Breast Cancer: Insights from Network Pharmacology, Molecular Docking, MD Simulation, ADME-Toxicity and DFT Profiles

Appendix 1. List of the 129 compounds associated with breast cancer

Compound	Torsion	Heavy Atoms	MW	HBD	HBA
(-)-Catechin	1	21	290.27	5	6
(-)-Mucronulatol	3	22	302.32	2	5
(+)-Epicatechin	1	21	290.27	5	6
(+)-Vestitol	2	20	272.30	2	4
(3S)-violanone	3	23	316.31	2	6
(S)-rosmarinic acid	7	26	360.32	5	8
[4]-Gingerol	8	19	266.33	2	4
1-Phenylnaphthalene	1	16	204.27	0	0
2'-Hydroxyformononetin	2	21	284.26	2	5
3'-Methoxyglabradin	2	26	354.40	2	5
Alloimperatorin	2	20	270.28	1	4
Alnustone	6	20	262.35	0	1
Aloin B	3	30	418.39	7	9
alpha-Calacorene	1	15	200.32	0	0
Ammijin	4	29	408.40	4	9
Arctigenin	7	27	372.41	1	6
Ar-Curcumene	4	15	202.34	0	0
Arnottianamide	5	28	381.38	1	7
Artemetin	6	28	388.37	1	8
Aureusidin	1	21	286.24	4	6
Belamcandin	5	26	358.34	1	7
beta-Anhydroicaritin	2	27	368.38	2	6
Calycosin	2	21	284.26	2	5
Cardamonin	4	20	270.28	2	4
Chryseriol	2	22	300.26	3	6
Columbianetin acetate	3	21	288.30	0	5
Cuparene	1	15	202.34	0	0
Dehydrocorydaline	4	27	366.43	0	5
Dehydrotanshinone II A	0	22	292.33	0	3
Dihydrochelerythrine	2	26	349.38	0	5
Dihydronitidine	2	26	349.38	0	5
Diphenylcyclopropenone	2	16	206.24	0	1
Dipiperitylmagnolol	9	40	538.80	2	2
Encecalin	2	17	232.28	0	3
Eseramine	5	23	318.37	2	5

Eurycarpin A	3	25	338.35	3	5
Feralolide	3	25	344.32	4	7
Fraxetin	1	15	208.17	2	5
Galgravin	6	27	372.46	0	5
Gallocatechin	1	22	306.27	6	7
gamma-Tocotrienol	9	30	410.63	1	2
Gancaonin A	4	26	352.38	2	5
Gancaonin G	4	26	352.38	2	5
Genistein	1	20	270.24	3	5
Gingerenone A	9	26	356.41	2	5
Gingerenone B	10	28	386.44	2	6
Gingerenone C	8	24	326.39	2	4
Gingerol	10	21	294.39	2	4
Glabranin	3	24	324.37	2	4
Glabrene	1	24	322.36	2	4
Glepidotin A	3	25	338.35	3	5
Glyasperin C	4	26	356.41	3	5
Glycyrin	5	28	382.41	2	6
Glycyrol	3	27	366.36	2	6
Glypallichalcone	5	21	284.31	1	4
Glyzaglabrin	1	22	298.25	2	6
Icaritin	4	27	368.38	3	6
Inflacoumarin A	3	24	322.36	2	4
Irigenin	4	26	360.32	3	8
Irisflorentin	5	28	386.35	0	8
Iristectorigenin A	3	24	330.29	3	7
Iristectorigenin B	3	24	330.29	3	7
Isogingerenone B	10	28	386.44	2	6
Isolicoflavonol	3	26	354.35	4	6
Isoolivil	5	27	376.40	5	7
Isopimpinellin	2	18	246.22	0	5
Isosativan	3	21	286.32	1	4
Isotrifoliol	1	22	298.25	2	6
Jatrorrhizine	3	25	338.38	1	5
Kaempferide	2	22	300.26	3	6
Karanjin	2	22	292.29	0	4
Licoagrocarpin	3	25	338.40	1	4
Licoagroisoflavone	2	25	336.34	2	5
Licochalcone A	6	25	338.40	2	4
Licochalcone B	4	21	286.28	3	5
Licocoumarone	4	25	340.37	3	5

Licoflavonol	3	26	354.35	4	6
Licoisoflavone B	1	26	352.34	3	6
Licoricone	5	28	382.41	2	6
Lupiwighteone	3	25	338.35	3	5
Magnolol	5	20	266.33	2	2
Marmesin	1	18	246.26	1	4
Matsukaze lactone	3	26	350.32	0	6
Medicarpin	1	20	270.28	1	4
Methoxyisolariciresinol	6	28	390.43	4	7
Moracin B	3	21	286.28	2	5
Moracin C	3	23	310.34	3	4
Moracin D	1	23	308.33	2	4
Moracin H	2	25	338.35	2	5
Mulberrofuran A	7	29	392.49	2	4
Naringenin	1	20	272.25	3	5
Neocryptotanshinone	2	23	314.38	2	4
Neouralenol	3	27	370.35	5	7
Nodakenetin	1	18	246.26	1	4
Nodakenin	4	29	408.40	4	9
Noririsflorentin	4	27	372.33	1	8
Norlapachol	1	17	228.24	1	3
Nyasol	4	19	252.31	2	2
Odoratin	3	23	314.29	2	6
Palmatine	4	26	352.40	0	5
Phaseol	2	25	336.34	2	5
Phaseollidin	2	24	324.37	2	4
Phaseollinisoflavan	1	24	324.37	2	4
Pinocembrin	1	19	256.25	2	4
Piperitylhonokiol	7	30	402.57	2	2
Prunetin	2	21	284.26	2	5
Pyrocurzerenone	0	16	212.29	0	1
Quercetin 3-3 DE	3	24	330.29	3	7
Rhamnazin	3	24	330.29	3	7
Rhamnocitrin	2	22	300.26	3	6
Salidroside	5	21	300.30	5	7
Secoisolariciresinol	9	26	362.42	4	6
Semilicoisoflavone B	1	26	352.34	3	6
Sigmoidin B	3	26	356.37	4	6
Sophoradochromene	7	34	458.59	2	4
Sophoranone	7	34	460.60	2	4
Spinacetin	3	25	346.29	4	8

Tectorigenin	2	22	300.26	3	6
Toralactone	1	20	272.25	2	5
Tricin	3	24	330.29	3	7
Trihydroxychalcone	3	19	256.25	3	4
Uralene	4	28	384.38	4	7
Vanillic Acid	2	12	168.15	2	4
Veraguensin	6	27	372.46	0	5
Vestitol, (-)-	2	20	272.30	2	4
Wedelolactone	1	23	314.25	3	7
Wighteone	3	25	338.35	3	5
Xanthorrhizol	4	16	218.34	1	1

Appendix 2. Docking result of 1XKK (Top 15 hits)

Ligand	MolDock Score	Rerank Score	Interaction	HBond	LE1	LE3	Docking Score	Total Score
Dipiperitylmagnolol	-134.26	-107.12	-154.11	0.00	-3.36	-2.68	-90.64	-492.17
Sophoranone	-126.35	-99.35	-145.11	-2.50	-3.72	-2.92	-85.52	-465.46
(S)-rosmarinic acid	-118.66	-110.22	-149.03	-7.11	-4.56	-4.24	-63.14	-456.96
Sophoradochromene	-116.36	-97.28	-138.61	-1.73	-3.42	-2.86	-91.35	-451.61
gamma-Tocotrienol	-111.94	-96.90	-133.26	0.00	-3.73	-3.23	-91.03	-440.10
Alnustone	-108.90	-96.83	-130.82	-0.41	-5.44	-4.84	-88.70	-435.95
Mulberrofuran A	-115.53	-96.61	-132.49	-3.24	-3.98	-3.33	-79.52	-434.70
Piperitylhonokiol	-116.36	-96.42	-132.15	0.00	-3.88	-3.21	-82.17	-434.21
Eurycarpin A	-108.96	-100.73	-130.56	-2.87	-4.36	-4.03	-80.55	-432.05
Moracin D	-114.79	-95.38	-122.09	-4.60	-4.99	-4.15	-77.57	-423.57
Inflacoumarin A	-107.70	-89.53	-120.79	-3.28	-4.49	-3.73	-83.94	-413.46
Glabrene	-106.43	-92.24	-122.38	-2.50	-4.43	-3.84	-79.12	-410.95
Moracin H	-108.68	-93.34	-119.24	-4.19	-4.35	-3.73	-76.60	-410.13
Isogingerenone B	-100.06	-87.07	-131.60	-8.03	-3.57	-3.11	-73.13	-406.57
Sigmoidin B	-100.11	-89.82	-120.47	-5.01	-3.85	-3.45	-81.66	-404.37

Appendix 3. Docking result of 3S7S (Top 15 hits)

Ligand	MolDock Score	Rerank Score	Interaction	HBond	LE1	LE3	Docking Score	Total Score
Dipiperitylmagnolol	-139.87	-92.44	-161.23	-1.95	-3.50	-2.31	-93.97	-495.26
Sophoranone	-141.58	-101.14	-157.93	-0.11	-4.16	-2.97	-85.97	-493.86
Sophoradochromene	-125.31	-105.94	-144.35	-2.74	-3.69	-3.12	-88.52	-473.67
Licoagroisoflavone	-117.25	-105.32	-142.65	-2.91	-4.69	-4.21	-77.99	-455.02
Piperitylhonokiol	-117.06	-98.49	-136.76	-1.15	-3.90	-3.28	-89.66	-450.30
gamma-Tocotrienol	-116.20	-94.50	-135.38	-0.49	-3.87	-3.15	-86.73	-440.32
Moracin H	-115.50	-99.91	-128.74	-0.33	-4.62	-4.00	-82.76	-435.85
Phaseol	-117.11	-95.61	-126.91	-2.81	-4.68	-3.82	-73.82	-424.77
Dihydronitidine	-110.81	-93.13	-128.57	-2.75	-4.26	-3.58	-78.31	-421.42
Phaseollinisoflavan	-111.19	-91.14	-127.99	-7.63	-4.63	-3.80	-73.10	-419.47
Mulberrofuran A	-113.54	-90.64	-122.62	-3.91	-3.92	-3.13	-78.45	-416.20
Sigmoidin B	-102.87	-94.90	-129.80	-1.70	-3.96	-3.65	-76.96	-413.83
Isogingerenone B	-104.43	-88.31	-133.67	-2.88	-3.73	-3.15	-74.34	-410.50
Icaritin	-99.77	-93.63	-131.60	-4.78	-3.70	-3.47	-72.68	-409.62
Neouralenol	-102.82	-93.74	-125.43	-7.72	-3.81	-3.47	-70.37	-407.36

Appendix 4. Docking result of 7PG6 (Top 15 hits)

Ligand	MolDock Score	Rerank Score	Interaction	HBond	LE1	LE3	Docking Score	Total Score
Dipiperitylmagnolol	-122.40	-97.34	-142.73	-0.53	-3.06	-2.43	-85.34	-453.84
Arctigenin	-118.26	-96.01	-137.18	-4.31	-4.38	-3.56	-63.47	-427.17
Gingerenone A	-106.21	-94.30	-131.89	-6.26	-4.08	-3.63	-73.28	-419.64
Phaseol	-109.68	-92.51	-118.41	-2.80	-4.39	-3.70	-84.65	-416.14
Gingerenone B	-100.31	-85.14	-131.82	-8.25	-3.58	-3.04	-81.23	-413.37
Gingerenone C	-101.92	-84.19	-124.66	-2.64	-4.25	-3.51	-81.67	-402.83
Lupiwighteone	-94.31	-88.60	-120.68	-7.09	-3.77	-3.54	-81.21	-399.21
Isolicoflavonol	-101.48	-90.81	-124.78	-2.50	-3.90	-3.49	-71.91	-398.87
Wedelolactone	-102.64	-88.53	-115.47	-6.39	-4.46	-3.85	-75.29	-396.64
Glycyrol	-106.52	-90.92	-118.34	-3.52	-3.95	-3.37	-69.97	-396.58
Semilicoisoflavone B	-93.85	-86.81	-119.88	-9.79	-3.61	-3.34	-77.60	-394.88
Dihydronitidine	-101.11	-85.47	-121.11	-2.39	-3.89	-3.29	-72.91	-390.15
Moracin D	-101.55	-86.36	-111.51	-2.99	-4.42	-3.75	-76.86	-387.44
Glyasperin C	-100.54	-85.47	-121.84	-5.18	-3.87	-3.29	-64.81	-385.00
Piperitylhonokiol	-96.50	-83.01	-118.83	0.00	-3.22	-2.77	-78.27	-382.59

Appendix 5. Plant source of the Traditional Chinese Medicine

Botanical	Compound
Ephedra herba	(S)-rosmarinic acid
Glechomae herba	(S)-rosmarinic acid
Menthae herba	(S)-rosmarinic acid
Perilla frutescens	(S)-rosmarinic acid
Schizonepetae herba	(S)-rosmarinic acid
Forsythiae fructus	Arctigenin
Fructus arctii	Arctigenin
Trachelospermum jasminoides	Arctigenin
Wikstroemiae indicae	Arctigenin
Magnolia biondii	Dipiperitylmagnolol
Magnolia grandiflora	Dipiperitylmagnolol
Magnolia officinalis	Dipiperitylmagnolol
Avena sativa	gamma-Tocotrienol
Bixa orellana	gamma-Tocotrienol
Carthami flos	gamma-Tocotrienol
Elaeis guineensis	gamma-Tocotrienol
Hordeum vulgare	gamma-Tocotrienol
Triticum aestivum	gamma-Tocotrienol
Zingiber officinale	Gingerenone A
Zingiberis rhizoma	Gingerenone A
Glycine max	Gingerenone B
Zingiber officinale	Gingerenone B
Zingiberis rhizoma	Gingerenone B
Crotalaria lachnophora	Licoagroisoflavone
Glycyrrhiza aspera	Licoagroisoflavone
Glycyrrhiza glabra	Licoagroisoflavone
Glycyrrhiza inflata	Licoagroisoflavone
Glycyrrhiza pallidiflora	Licoagroisoflavone
Dolichos trilobus	Phaseol
Phaseolus acutifolius	Phaseol
Phaseolus vulgaris	Phaseol
Vigna radiata	Phaseol
Magnolia biondii	Piperitylhonokiol
Magnolia grandiflora	Piperitylhonokiol
Magnolia obovate	Piperitylhonokiol
Magnolia officinalis	Piperitylhonokiol
Sophora flavescens	Sophoradochromene
Sophora japonica	Sophoradochromene
Sophorae tonkinensis	Sophoradochromene
Canthium subcordatum	Sophoranone
Euglena gracilis	Sophoranone
Gymnopus peronatus	Sophoranone

Millettia pulchra	Sophoranone
Pinus muricata	Sophoranone
Polygonum salicifolium	Sophoranone
Sloanea rhodantha	Sophoranone
Sophorae tonkinensis	Sophoranone
Zingiber amaricans	Sophoranone

Appendix 6. ADME-Toxicity Analysis of top 3 docking hits

Compounds	QED	AMES	BBB	Bioavailability	CYP1A2	CYP2C19	CYP2C9	Carcinogen	ClinTox
(S)-rosmarinic acid	0.298	0.12	0.39	0.42	0.14	0.24	0.21	0.07	0.14
Arctigenin	0.753	0.14	0.56	0.84	0.30	0.91	0.23	0.05	0.04
Dipiperitylmagnolol	0.312	0.18	0.31	0.65	0.07	0.45	0.46	0.68	0.03
Gingerenone A	0.668	0.23	0.38	0.60	0.64	0.93	0.29	0.14	0.03
Sophoradochromene	0.262	0.31	0.26	0.52	0.42	0.68	0.24	0.30	0.08
Sophoranone	0.425	0.25	0.21	0.64	0.19	0.64	0.24	0.32	0.05

Appendix 7. Toxicity Analysis of the top 3 docking hits

Compounds	SR-HSE	Skin_Reaction	hERG	Caco2	Half_Life	Hydration	LD50	Lipophi
(S)-rosmarinic acid	0.07	0.54	0.12	-6.34	-21.50	-11.90	1.77	-0.62
Arctigenin	0.14	0.52	0.53	-4.96	-29.50	-7.76	1.99	2.73
Dipiperitylmagnolol	0.71	0.75	0.77	-5.41	-22.27	-3.48	2.67	4.55
Gingerenone A	0.56	0.47	0.56	-5.00	-29.89	-8.03	1.69	3.53
Sophoradochromene	0.80	0.60	0.75	-5.24	46.87	-5.96	2.79	4.98
Sophoranone	0.71	0.50	0.62	-5.16	13.14	-5.34	2.96	4.76

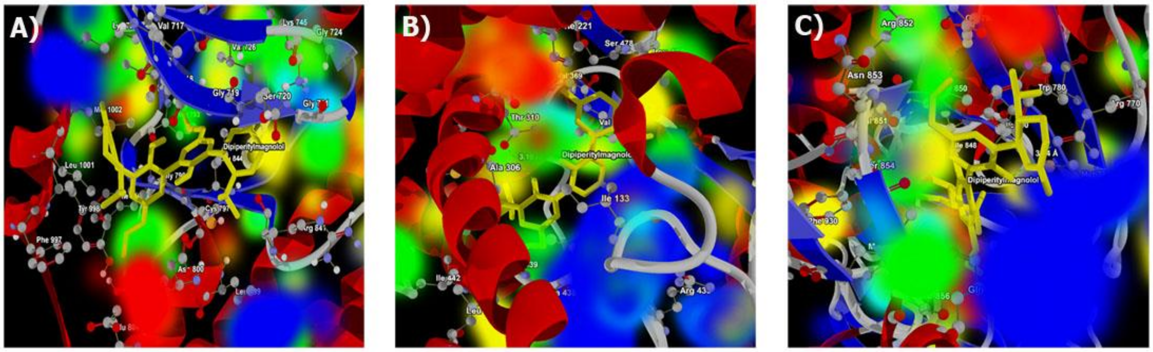

Appendix 8. Energy map analysis of the top docking hits (Dipiperitylmagnolol) at the active site of 1XKK, 3S7S and 7PG6 showing the HBA/HBD regions (yellow/turquoise) electro-positive/negative regions (red/blue)

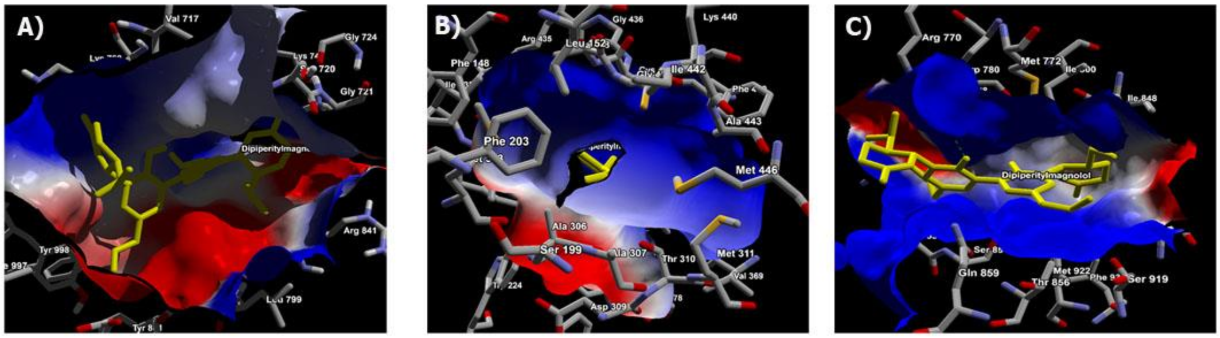

Appendix 9. Electrostatic interaction maps of the top docking hit (Dipiperitylmagnolol) at the active site of 1XKK, 3S7S, 7PG6.

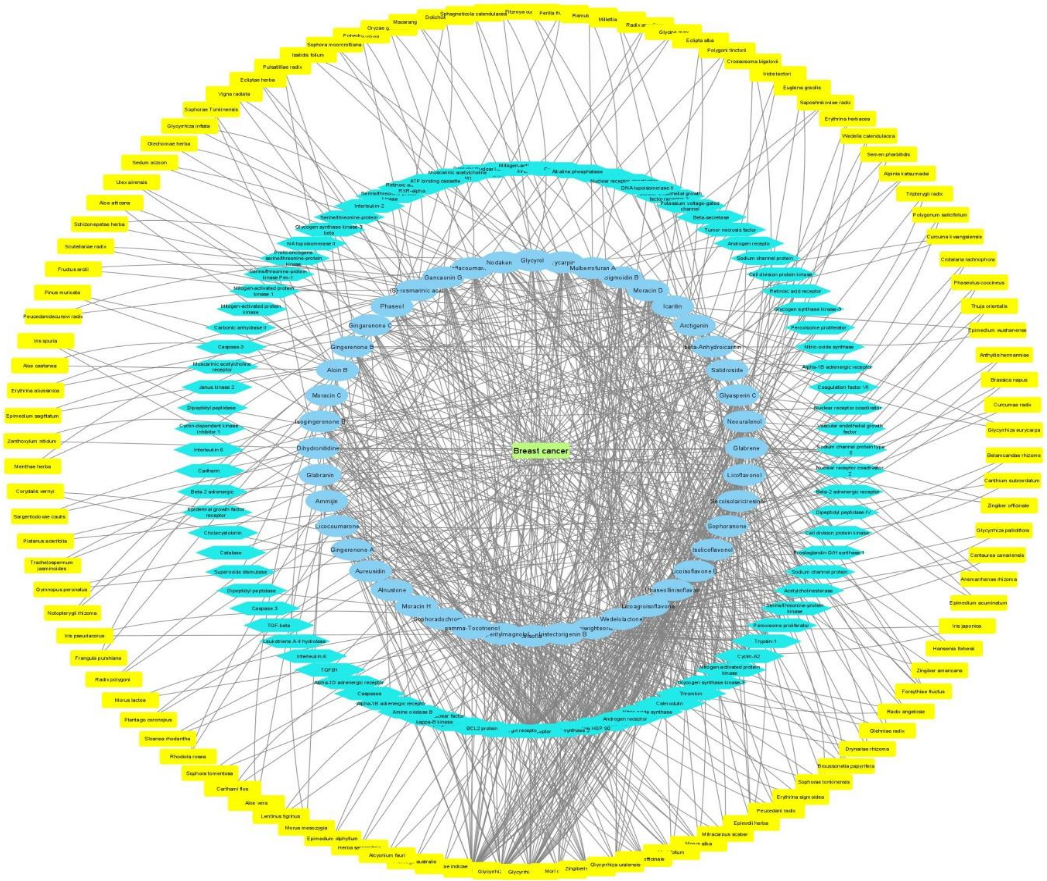

Appendix 10. Network pharmacology map of the investigated compounds and their target molecule and pathway showing the relationships between various elements associated with breast cancer. Light blue oval indicates compounds, hexagons cyan blue indicates target enzyme, yellow rectangles indicates herbal plants.

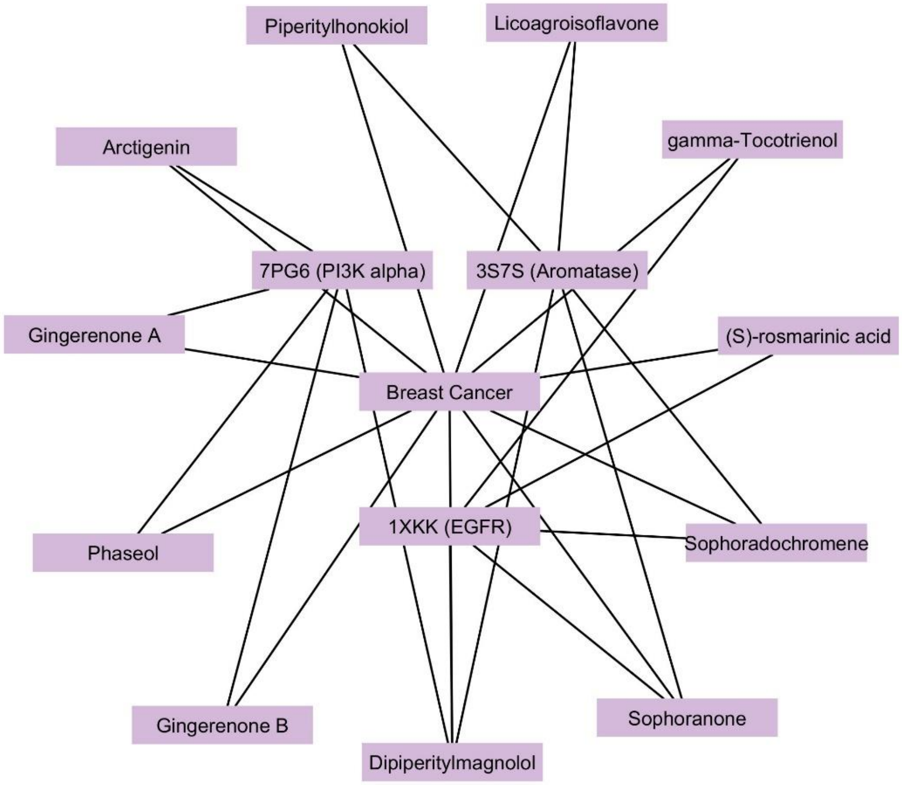

Appendix 11. Network pharmacology map of the top 3 docking hits of 1XKK, 7PG6 and 3S7S.

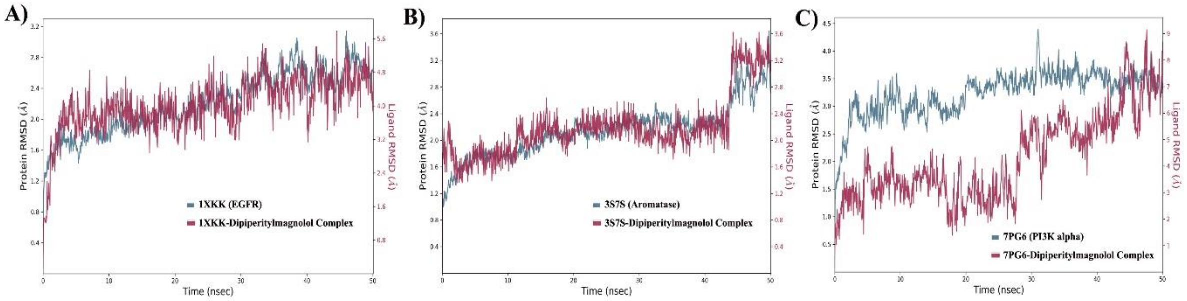

Appendix 12. RMSD Analysis of 1XKK-Dipiperitylmagnolol (Fig. 8A) 3S7S-Dipiperitylmagnolol (Fig. 8B) and 7PG6-Dipiperitylmagnolol (Fig. 8C) during the 50 ns MD production.

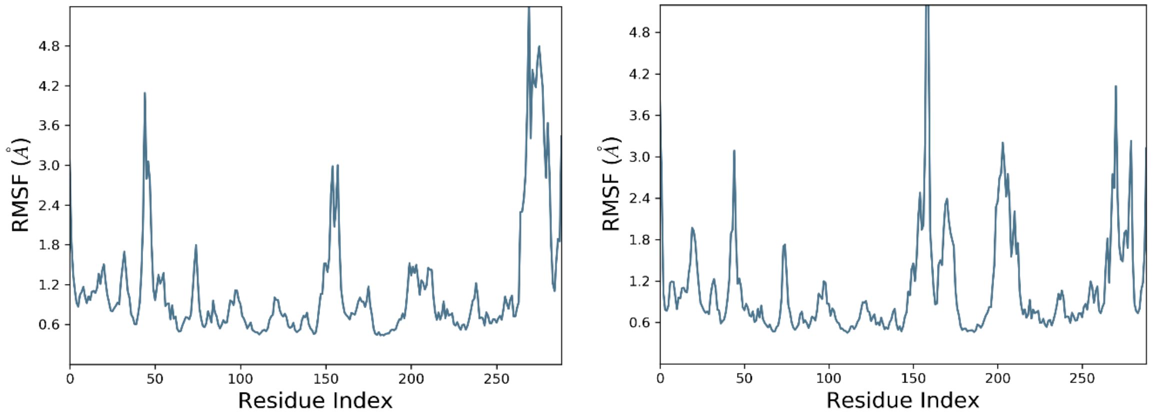

(A) **(B)**

Appendix 13. RMSF of (A) 1XKK and (B) 1XKK-Dipiperitylmagnolol complex

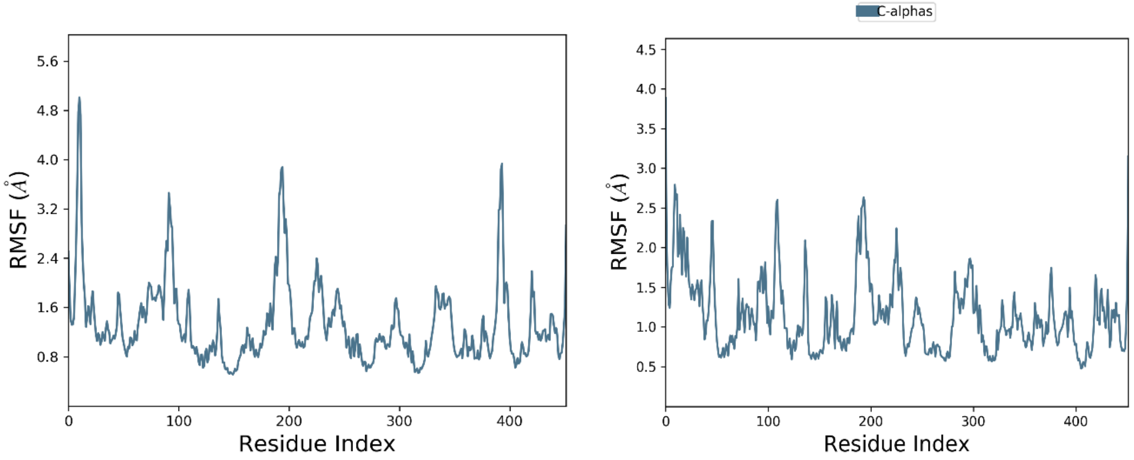

(A) **(B)**

Appendix 14. RMSF of (A) 3S7S and (B) 3S7S-Dipiperitylmagnolol complex

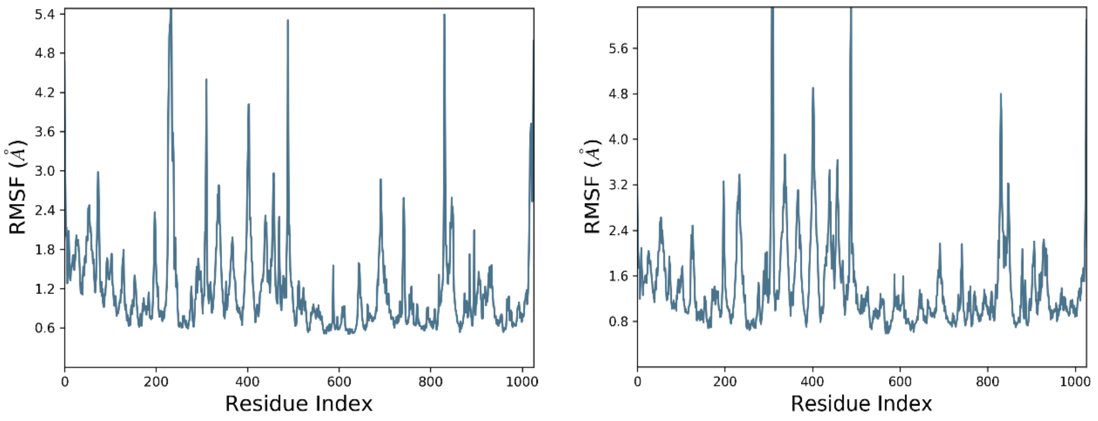

(A) **(B)**

Appendix 15. RMSF of (A) 7PG6 and (B) 7PG6-Dipiperitylmagnolol complex

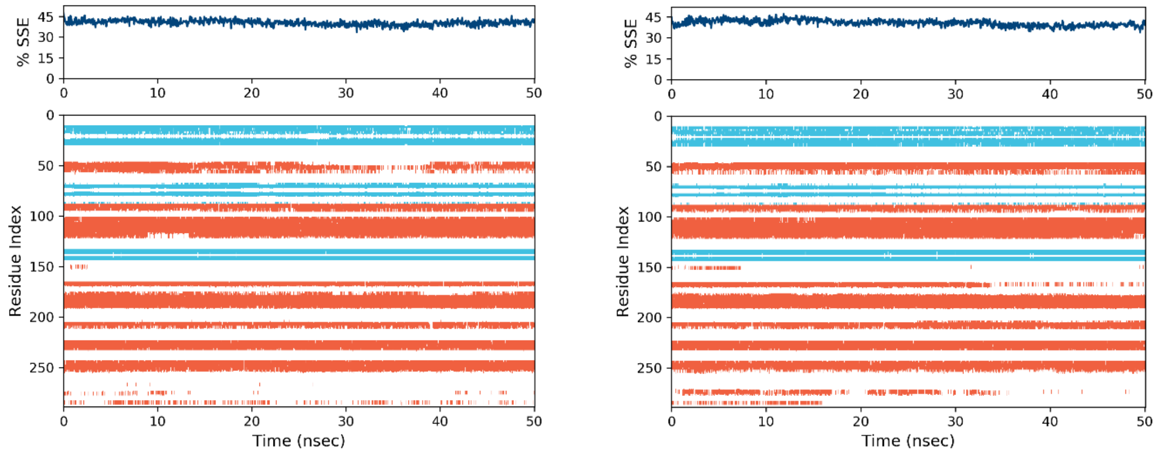

(A) **(B)**

Appendix 16. Protein secondary structure elements (SSE) like alpha-helices and beta-strands are monitored throughout the 50 ns simulation for (A) 1XKK and (B) 1XKK-Dipiperitylmagnolol complex.

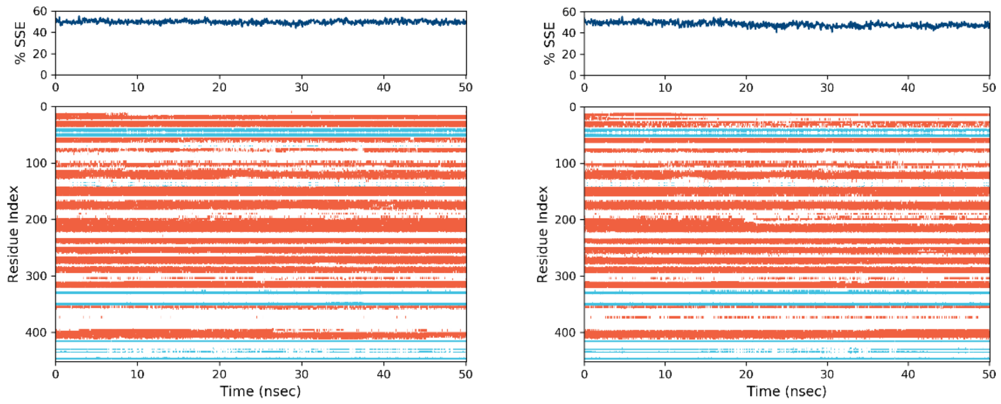

(A) **(B)**

Appendix 17. Protein secondary structure elements (SSE) like alpha-helices and beta-strands are monitored throughout the 50 ns simulation for (A) 3S7S and (B) 3S7S-Dipiperitylmagnolol complex.

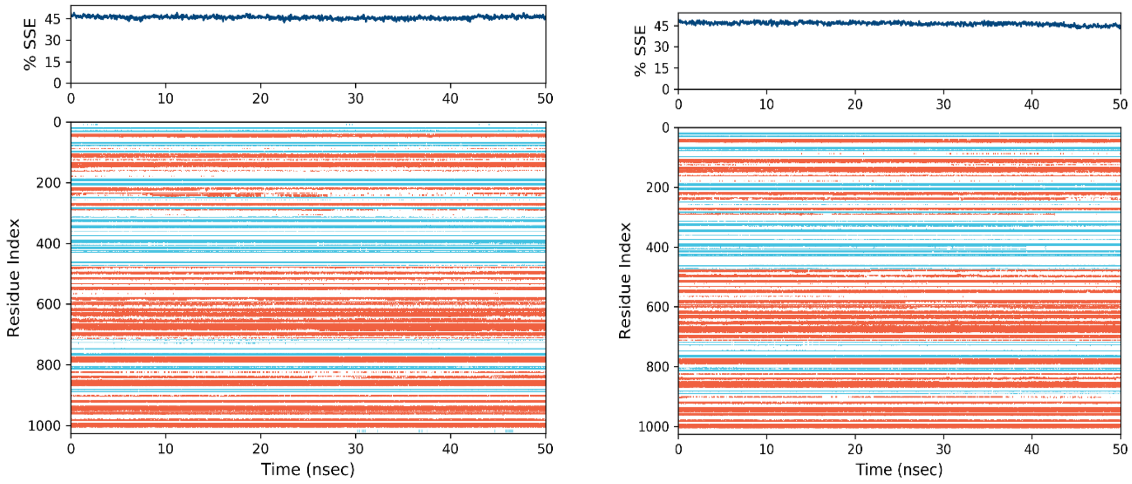

(A) **(B)**

Appendix 18. Protein secondary structure elements (SSE) like alpha-helices and beta-strands are monitored throughout the 50 ns simulation for (A) 7PG6 and (B) 7PG6-Dipiperitylmagnolol complex.

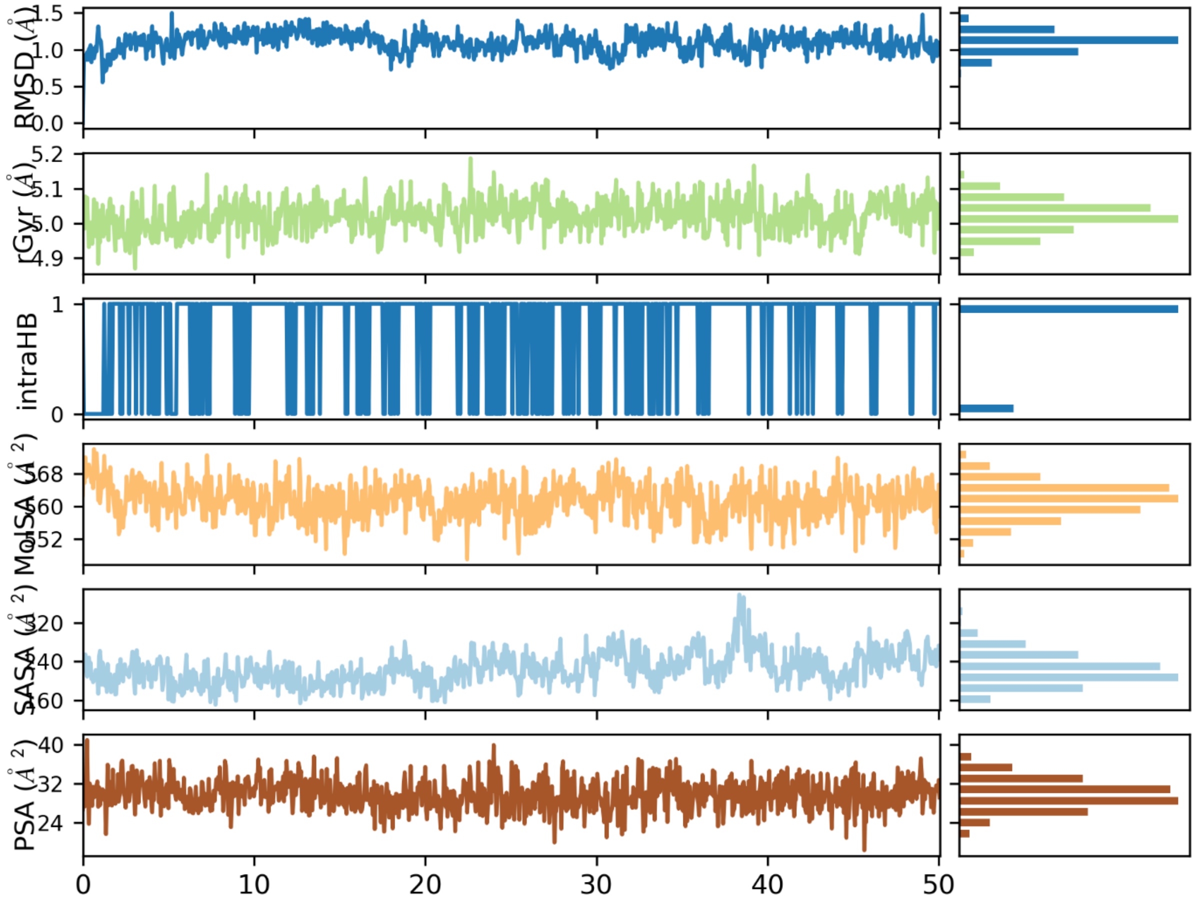

Appendix 19. Ligand interaction profile of 1XKK-Dipiperitylmagnolol complex

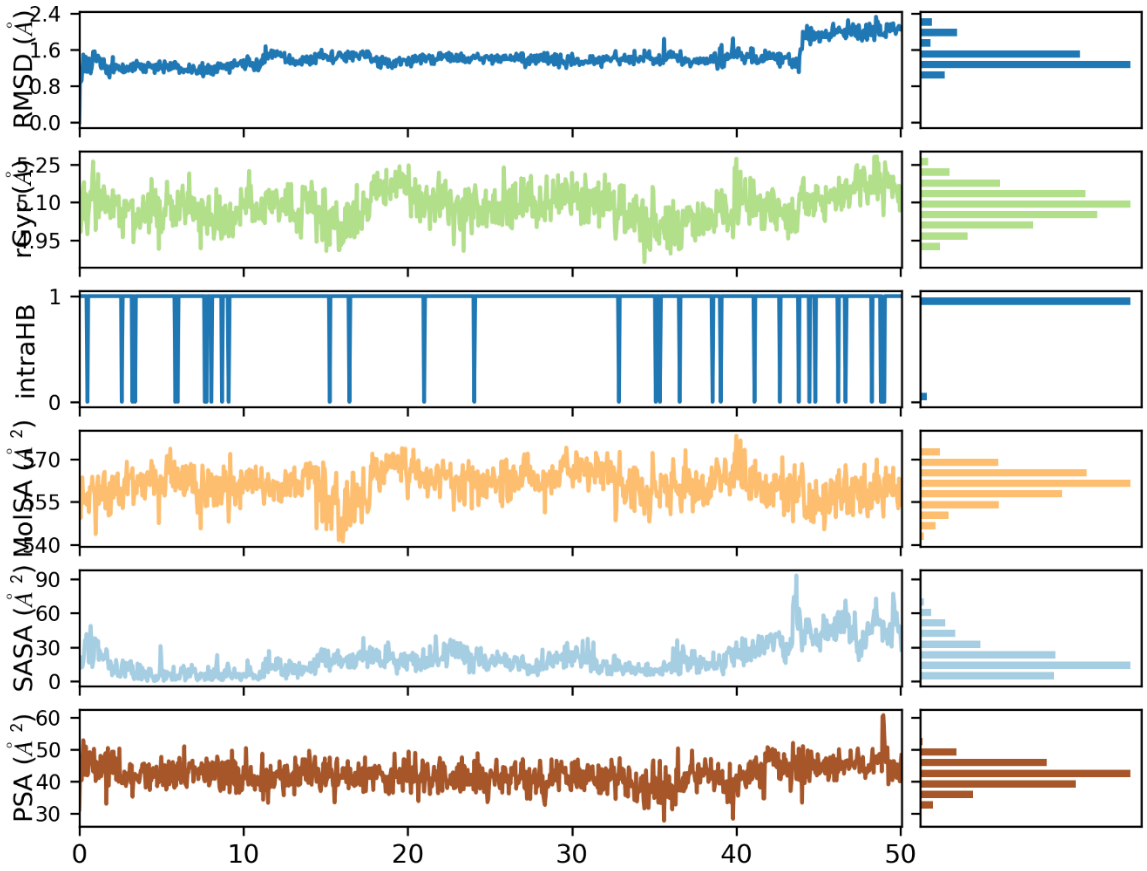

Appendix 20. Ligand interaction profile of 3S7S-Dipiperitylmagnolol complex

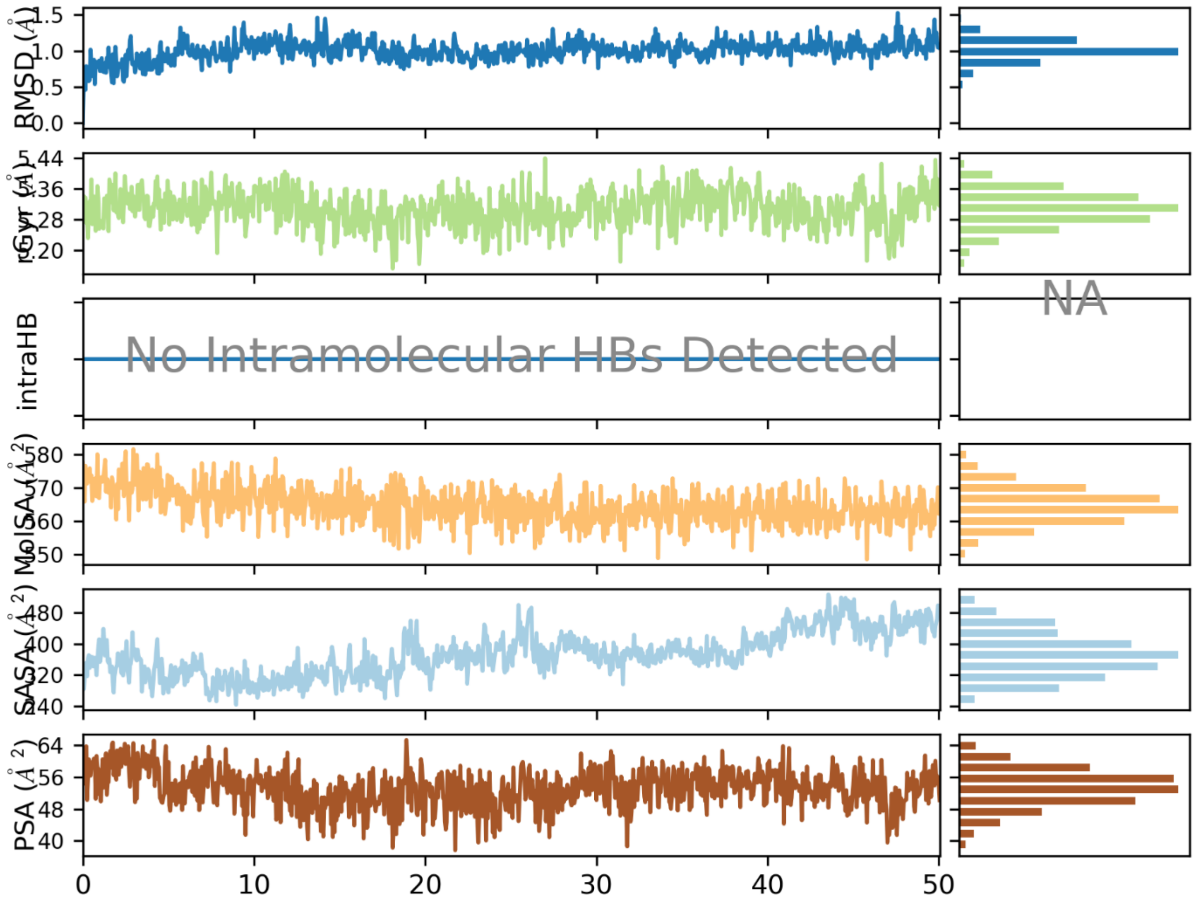

Appendix 21. Ligand interaction profile of 7PG6-Dipiperitylmagnolol complex

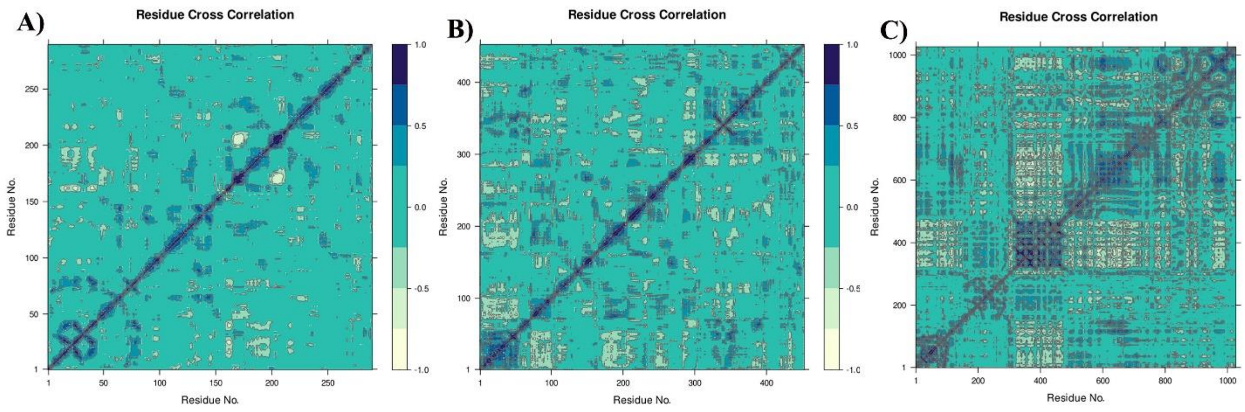

Appendix 22. DCCM Plot analysis of 1XKK-Dipiperitylmagnolol (Fig. 9A) 3S7S-Dipiperitylmagnolol (Fig. 9B) and 7PG6-Dipiperitylmagnolol (Fig. 9C) during the 50 ns MD production.

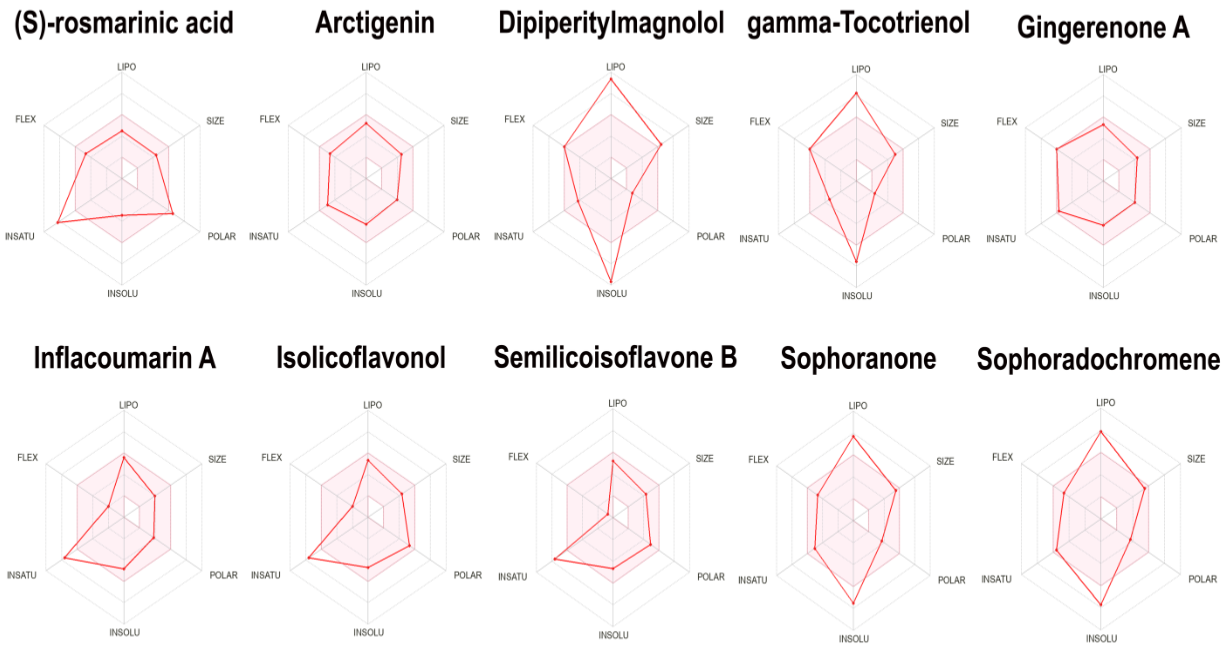

Appendix 23. ADME-Toxicity radar map of the top 3 docking hits of each of the target proteins.

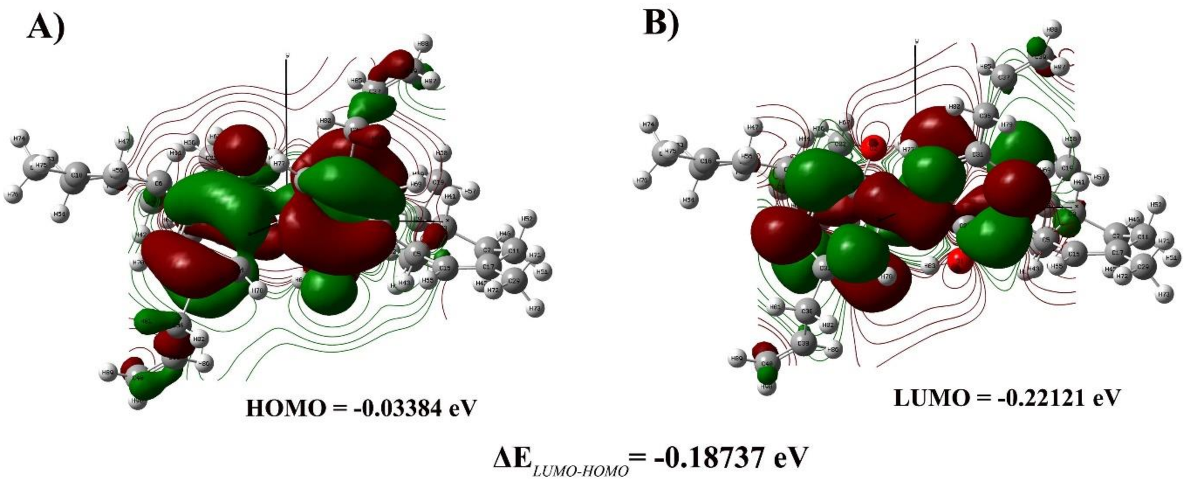

Appendix 24. (A) HOMO and (B) LUMO energies of Dipiperitylmagnolol
